# Atorvastatin-mediated rescue of cancer-related cognitive changes in combined anticancer therapies

**DOI:** 10.1371/journal.pcbi.1009457

**Published:** 2021-10-20

**Authors:** Junho Lee, Jin Su Kim, Yangjin Kim

**Affiliations:** 1 Department of Mathematics, Konkuk University, Seoul, Republic of Korea; 2 Radiological and Medico-Oncological Sciences, University of Science and Technology, Seoul, Republic of Korea; 3 Division of RI Application, Korea Institute Radiological and Medical Sciences, Seoul, Republic of Korea; 4 Department of neurosurgery, Brigham and Women’s Hospital & Harvard Medical School, Boston, Massachusetts, United States of America; University of Southern California, UNITED STATES

## Abstract

Acute administration of trastuzumab (TZB) may induce various forms of cognitive impairment. These cancer-related cognitive changes (CRCC) are regulated by an adverse biological process involving cancer stem cells (CSCs) and IL-6. Recent studies have reported that atorvastatin (ATV) may change the dynamic of cognitive impairment in a combination (TZB+ATV) therapy. In this study, we investigate the mutual interactions between cancer stem cells and the tumor cells that facilitate cognitive impairment during long term TZB therapy by developing a mathematical model that involves IL-6 and the key apoptotic regulation. These include the densities of tumor cells and CSCs, and the concentrations of intracellular signaling molecules (NF*κ*B, Bcl-2, BAX). We apply the mathematical model to a single or combination (ATV+TZB) therapy used in the experiments to demonstrate that the CSCs can enhance CRCC by secreting IL-6 and ATV may interfere the whole regulation. We show that the model can both reproduce the major experimental observation on onset and prevention of CRCC, and suggest several important predictions to guide future experiments with the goal of the development of new anti-tumor and anti-CRCC strategies. Moreover, using this model, we investigate the fundamental mechanism of onset of cognitive impairment in TZB-treated patients and the impact of alternating therapies on the anti-tumor efficacy and intracellular response to different treatment schedules.

## Author summary

A conventional drug, trastuzumab (TZB), was shown to be an effective weapon in killing cancer cells in brain. However, long term treatment of TZB increases the proportion of cancer stem cells (CSCs) in the tumour microenvironment (TME) and induces up-regulation of pro-tumoral molecules such as IL-6 in TME. These cancer cells then become more resistant to this chemotherapy through the IL-mediated up-regulation of NF*κ*B and CSCs. More importantly, these changes in TME result in a serious side effect, cognitive impairment called *cancer-related cognitive changes (CRCC)*. The detailed mechanism of CRCC is still poorly understood. However, cancer patients with chemotherapy-induced cognitive impairment can have long-term or delayed mental changes. In this study, we investigated the fundamental mechanism of CRCC in cancer patients based on experiments and a mathematical model that describes how tumor cells interact with CSCs in response to chemo drugs. In particular, we investigate how TZB-induced CSCs with modified IL-6 landscapes shape the cognitive functions in cancer patients. We showed that the combination treatment with another drug, atorvastatin (ATV), can abrogate the TZB-induced CRCC and enhance the survival probability of cancer patients by synergistic anti-tumor effect. We demonstrate that the cognitive functions and survival rates in cancer patients depend on the apoptotic signaling pathways via the critical communication and IL-6 landscapes of stimulated CTCs.

## Introduction

A receptor tyrosine-protein kinase erbB-2 also known as epidermal growth factor receptor 2 (HER2) is over-expressed in many cancers including breast, ovarian, and gastric cancers, and heterodimerization of this initiates various signaling pathways leading to aggressive proliferation and tumorigenesis [1], thus becoming a well-known therapeutic target in cancer research over 30 years [2]. HER2 plays an important role in regulation of complex intracellular pathways in response to biochemical stimuli such as epidermal growth factor (EGF) and transforming growth factor (TGF-*β*) for critical decision of cell fate within cancer cells [3–6]. Trastuzumab (TZB), the first humanized mono-clone antibody, selectively targets HER2 on cancer cells and provides successful survival benefits in HER2^+^ metastatic breast cancers [7, 8]. It has been shown that TZB initiates the apoptosis signaling in cancer cells by suppressing the anti-apoptotic family, Bcl-2 members [9], which subsequently induces up-regulation of BAX, apoptosis inducer. However, the long term treatment of TZB can cause cancer-related cognitive changes (CRCC) [10], sometimes referred to as ‘chemo-brain’, as a side effect. Even if TZB is a popular choice of treatment in breast cancers, anti-tumor efficacy of TZB has been investigated in various cancers including brain cancers [11, 12], showing different survival rates [10]. How TZB treatment in organs is regulated for CRCC in brain is poorly understood [10]. In addition, this long term chemotherapy can also enhance proliferation of cancer stem cells (CSCs) that secrete more than 100 times more IL-6 than parent cells [13], which induces up-regulation of NF-*κ*B to support anti-apoptosis, reducing anti-tumor efficacy [14]. CSCs have been identified in many cancers including brain cancer [15, 16], breast cancer [17] and melanoma [18] with their pro-tumorigenic characteristics of enhancement of tumor growth, post-injury regeneration and metastasis to other organs [19].

Despite the long history of CRCC and research, the exact biological mechanism is still poorly understood [20, 21]. Coello *et al*. [22] suggested that up-regulation of IL-6 levels after long TZB treatment is a major cause of CRCC. Various studies suggest that cognitive impairment may be associated with the interaction of various cytokines, including IL-6, IL-1*β*, TNF-*α*, and microglia [23–25]. IL-6 is a pleiotropic cytokine that can enhance proliferation of cancer cells. Despite beneficial effects of IL-6 on neuronutrient properties and central nervous system (CNS), its over-expression induces genetically harmful results, adding to the pathological physiology associated with CNS disorders. Cognitive symptoms associated with IL-6 include key clinical manifestations of dementia and other systemic pathology (such as liver cirrhosis, cardiovascular disease, etc.) [26].

Statins, well-known HMG-CoA reductase inhibitors, are widely used to suppress cholesterol levels by curbing HMG-CoA enzymes [27, 28]. Recent studies showed that statins can mediate anti-proliferative, pro-apoptotic and anti-invasive functions in various cancer cells [29–31]. Atorvastatin (ATV), one of this kind, was shown to accelerate radio-sensitivity and cell death of prostate cancer cells [32]. In addition, ATV treatment was shown to induce attenuation of cognitive impairment at the cellular level in mice [33, 34]. Furthermore, ATV infusion was able to suppress IL-6 levels [10].

In a recent experimental study, Lee *et al*. [10] suggested that ATV injection during TZB treatment can prevent CRCC and enhance anti-tumor effect of TZB. In this paper, we develop a mathematical model of the tumor growth and CRCC in the absence and presence of a combination (TZB+ATV) therapy. We consider an intracellular network in apoptosis pathways involving NF-*κ*B, Bcl-2 and BAX. We show that the mathematical model can reproduce many of the experimental observations on TZB-induced CRCC and we make predictions as to how combination therapy can affect the outcome.

## Materials and methods

**Fig 1:**
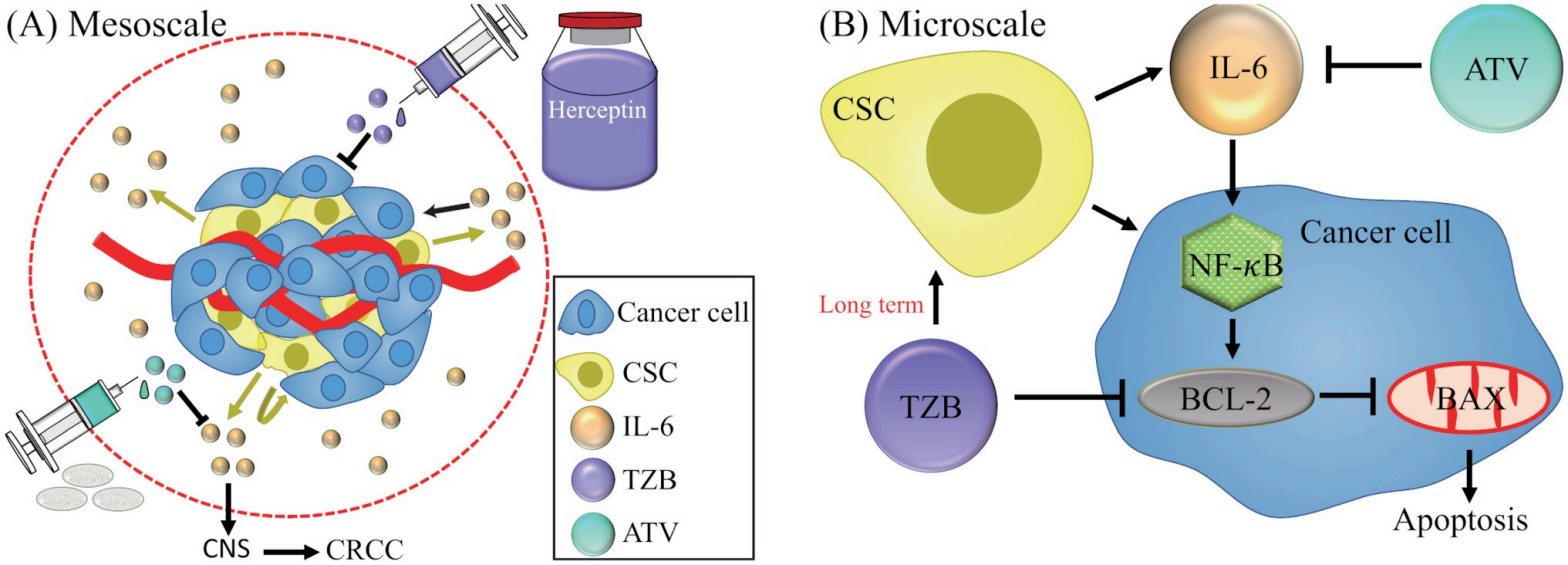
A conceptual flowchart involving anti-cancer (ATV) therapy and CSC-induced CRCC at meso- (A) and micro-scale (B) in TZB therapy [10]. Arrows indicate induction and activation while hammerheads indicate inhibition.

In this section, we develop a mathematical model based on key variables in the schematic diagram in Fig 1. We introduced the following variables:

$$\begin{array}{ccc} & & F(t)=\text{concentration}\;\text{of}\;\text{NF}\kappa \text{B}\;\text{at}\;\text{time}\;t\;(\mu M),\\ & & B(t)=\text{concentration}\;\text{of}\;\text{Bcl-2}\;\text{at}\;\text{time}\;t\;(\mu M),\\ & & X(t)=\text{concentration}\;\text{of}\;\text{BAX}\;\text{at}\;\text{time}\;t\;(\mu M),\\ & & C\left(t\right)=\text{density}\;\text{of}\;\text{cancer}\;\text{cells}\;\text{at}\;\text{time}\;t\;\left(cells/cm^{3}\right),\\ & & S\left(t\right)=\text{density}\;\text{of}\;\text{cancer}\;\text{stem}\;\text{cells}\;\text{at}\;\text{time}\;t\;\left(cells/cm^{3}\right),\\ & & L\left(t\right)=\text{concentration}\;\text{of}\;\text{IL-6}\;\text{at}\;\text{time}\;t\;\left(g/cm^{3}\right), \end{array}$$

### Intracellular network

**Fig 2:**
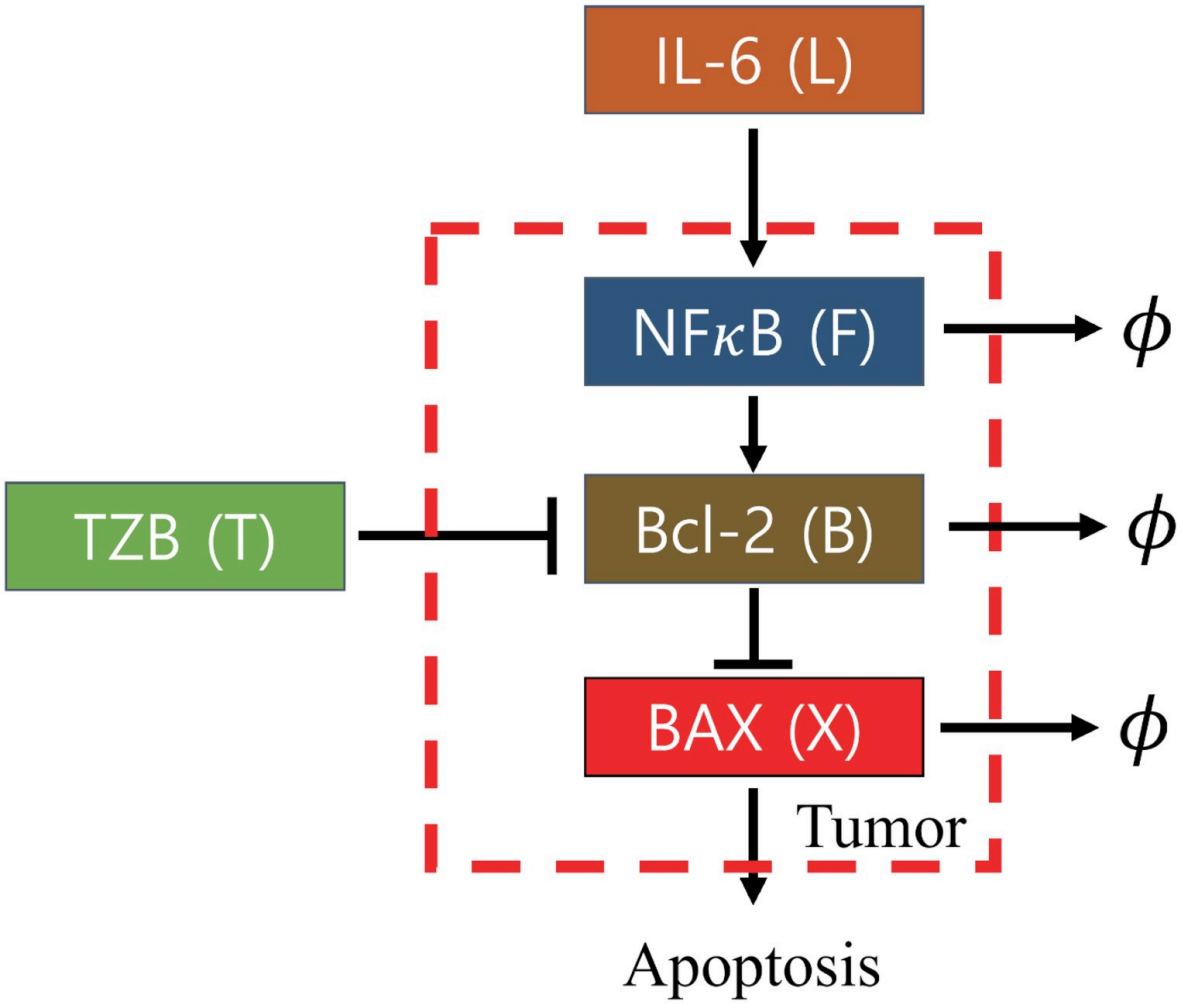
Conceptual model of regulation of intracellular variables (NF*κ*B, Bcl-2, and BAX) with extracellular stimuli (IL-6 and TZB) in the tumor cell apoptosis process [43].

In order to incorporate the role of apoptosis pathways in regulation of tumor cell killing by TZB [10], we developed a mathematical network of the NF*κ*B-Bcl2-BAX system based on experimental observations [10] in Fig 2. The scheme includes signaling supply, autocatalytic activities, protein degradation of those key molecules, inhibition of the Bcl-2 by TZB, and inhibition of BAX by Bcl-2. While IL-6 induces NF*κ*B activities [35], forming a positive feedback [36], TZB kills tumor cells by inducing apoptosis through inhibition of Bcl-2 [9], leading to up-regulation of BAX. When activated, NF-*κ*B is converted into nucleus to activate various genes, including Bcl-2, which in turn inhibits the apoptosis gene BAX [37–41]. BAX is a well-known apoptosis-inducer [42, 43] while Bcl-2 inhibits BAX activities [43]. In particular, activation of an IL-6 Inflammatory signaling was shown to mediate TZB resistance in HER2^+^ cancers through expansion of the CSC population [13]. IL-6 increases the level of NF-*κ*B [14] which in turn induces Bcl-2, anti-apoptotic family [44].

In this work, we derive governing equations of given intracellular variables **z** = (*z*_1_, *z*_2_, …, *z*_*N*_) based on the mass balance equations of the form:

$$\begin{array}{ccc} & & \frac{dz_{i}}{dt}=\underset{source}{\underset{︸}{f_{i}\left(z\right)}}+\underset{inhibition}{\underset{︸}{g_{i}\left(z\right)}}-\underset{decay}{\underset{︸}{h_{i}\left(z\right)}},\;\;\left(i=1,\ldots ,N\right) \end{array}$$ (1)

where the functions *f*_*i*_(**z**), *g*_*i*_(**z**) and *h*_*i*_(**z**) represent the source, inhibition, and decay/degradation due to natural decay, respectively. We take a simple form of the decay process, *h*_*i*_(**z**) = *μ*_*i*_ *z*_*i*_ with a decay rate *μ*_*i*_. Various forms of the source term *f*_*i*_(**z**) can be taken based on biological observations in general. We take a fractional form [45–48]

$$\begin{array}{c} g_{i}\left(z\right)=\frac{\eta_{1}\eta_{2}^{n}}{\eta_{2}^{n}+\alpha_{i}F\left(z_{j}\right)} \end{array}$$ (2)

to represent autocatalytic activity with the negative feedback of the intracellular variable *z*_*i*_ by inhibitory intracellular variable *z*_*j*_ (*i* ≠ *j*) in Eq (1). Here, *η*_1_, *η*_2_ are Hill-type constants. A function *F*(*z*_*i*_) and the inhibition strength parameter *α*_*i*_ represent the inhibition process of the variable *z*_*j*_ ($\mu_{i},\eta_{1},\eta_{2},\alpha_{i}\in R^{+}$, $n\in Z^{+}$). *F*(*z*_*i*_) can be a linear or non-linear function, which determines the strength and dynamics of negative feedbacks. In the absence and presence of source, this inhibition term in Eq (2) provides the baseline level ($z_{i}^{*}\approx \frac{\eta_{1}}{\mu_{i}}$ and $z_{i}^{*}\approx \frac{f_{i}+\eta_{1}}{\mu_{i}}$) of the given variable *z*_*i*_ at equilibrium when the inhibition function *F*(*z*_*j*_) is zero or small. When *F*(*z*_*j*_) is large, we get the down-regulated level, $z_{i}^{*}=\frac{\eta_{1}}{\mu_{i}}\epsilon$ and $z_{i}^{*}=\frac{f_{i}+\eta_{1}\epsilon }{\mu_{i}}$ in the absence and presence of source, respectively (S1 Text). Here, $\epsilon =\frac{\eta_{2}^{n}}{\eta_{2}^{n}+\alpha_{i}F\left(z_{j}\right)})⪡1$. Thus, given decay rate, the balance between the source and strength of the negative feedback from *z*_*j*_ can determine the level of the variable *z*_*i*_. By comparing the up- or down-regulated concentration of *z*_*i*_ with empirical data in response to the inhibitory module (*F*(*z*_*j*_)) in the system, one can construct a linear or non-linear form of *F*(*z*_*j*_) and estimate parameter values of a mathematical model in Eqs (1) and (2). Other studies [45–48] have shown that this fractional form may regenerate analytic structure of positive and negative feedbacks in an intracellular network and provide qualitative nonlinear dynamics such as bi-stability with experimental validation. For example, the bi-stable system from the inhibition of the protein module (Myc and the E2Fs) by the miR-17 cluster in a fraction form allowed authors to conceptually define the *cancer zone* and explain the dual role of miR-17s as oncogene and tumor suppressor in the framework of cell cycle [45]. Kim *et al*. [46] also showed that a mathematical model of intracellular regulators in a fraction form (2) successfully reproduced experimental observations such as the mutual inhibition between miR-451 and AMPK in response to various glucose conditions [49], and the nonlinear behavior such as bi-stability can explain complex behaviors among invasive or proliferative phenotypes in a tumor microenvironment. Other forms of negative feedbacks were used in the literature [6, 50] with the same goal of modeling the inhibition of key players but these introduce more variables due to detailed chemical reactions that the model is based on.

Based on this framework and biological observations, we can write the phenomenological equations for the rate change of concentrations (*μM*) of those key intracellular modules (*F*, *B*, *X*) as follows:

$$\begin{array}{ccc} & & \frac{dF}{dt}=f_{1}\left(L\right)-\mu_{F}F, \end{array}$$ (3)

$$\begin{array}{ccc} & & \frac{dB}{dt}=f_{2}\left(F\right)+\frac{k_{5}k_{4}^{2}}{k_{4}^{2}+\delta F_{1}\left(T\right)}-\mu_{B}B, \end{array}$$ (4)

$$\begin{array}{ccc} & & \frac{dX}{dt}=f_{3}+\frac{k_{7}k_{6}^{2}}{k_{6}^{2}+\gamma F_{2}\left(B\right)}-\mu_{X}X. \end{array}$$ (5)

where *f*_1_(*L*) is a positive function that represents the signaling source of NF*κ*B from the level of IL-6 (*L*), whose dynamics will be introduced below, and others to NF*κ*B, *f*_2_(*F*) is a positive function that represents the signaling source of Bcl-2 from NF*κ*B and others to Bcl-2, *f*_3_ is the signaling source of BAX, *k*_5_, *k*_7_ are the autocatalytic enhancement parameters for activities of Bcl-2 and BAX, respectively, *k*_4_, *k*_6_ are the inhibition constants, *δ* is the inhibition strength of Bcl-2 by TZB, *γ* is the inhibition strength of BAX by Bcl-2, and *μ*_*F*_, *μ*_*B*_ and *μ*_*X*_ are the decay rates of the NF-*κ*B, Bcl-2 and BAX, respectively. Here, *T* in the second term of Eq (4) represents the concentration of the injected TZB (*g*/*mm*^3^). Table 1 provides the specific values of parameters in Eqs (3)–(5) with appropriate units. As indicated in Eq (3), the IL-6 signal *L* increases the rate of NF*κ*B activation through the function *f*_1_(*L*) in the first term, satisfying $\frac{\partial f_{1}}{\partial L}>0$ for all non-negative *L*. In a similar fashion, up-regulation of NF*κ*B increases the rate of Bcl-2 activation through the function *f*_2_(*F*) in the second term ($\frac{\partial f_{2}}{\partial F}>0$ for all non-negative *F*) and the signaling *f*_3_ enhances BAX activities in the third term (*f*_3_ > 0). On the other hand, TZB-dependent inhibition of Bcl-2 activities is expressed through a function *F*_1_(*T*) in the denominator of the second term in Eq (4). A very simple requirement of this function is that $\frac{\partial F_{1}}{\partial T}>0$ for all positive *T*. Similarly, the Bcl-2-dependent suppression of BAX activity is explored through a function *F*_2_(*B*) in the denominator of the second term in Eq (5). The fractional form in Eq (2) was taken for the qualitative modeling of negative feedbacks of Bcl-2 in Eq (4) and BAX in Eq (5). Based on biological observations (Fig 2A), we can have several assumptions satisfying mathematical conditions above as follows:

$$\begin{array}{c} \begin{array}{cc} & f_{1}\left(L\right)=\lambda_{F}+\lambda_{2}L,\;\;f_{2}\left(F\right)=\lambda_{B}+\lambda_{3}F,\;\;f_{3}=\lambda_{X},\;\;F_{1}\left(T\right)=T^{2},\;\;F_{2}\left(B\right)=B^{2}, \end{array} \end{array}$$ (6)

where λ_*F*_, λ_*B*_ and λ_*X*_ represent the signaling source of the NF-*κ*B, Bcl-2 and BAX, respectively, λ_2_ is the activation rate of NF-*κ*B by IL-6, λ_3_ is the activation rate of Bcl-2 by NF-*κ*B. The simplest forms, linear expressions, were chosen for functions *f*_1_, *f*_2_ in Eq (6) while quadratic forms for functions *F*_1_, *F*_2_ are chosen for the higher sensitivity of inhibition of one variable by another variable. In particular, our choice of the quadratic forms of functions *F*_1_, *F*_2_ in the framework of negative feedbacks in Eqs (1) and (2) allows us to better fit the simulated concentrations of Bcl-2 and IL-6 to experimental data in various TZB and ATV conditions. With assumptions (6), we have the governing equations for NF*κ*B (*F*), Bcl-2 (*B*), BAX (*X*) as follows:

$$\begin{array}{ccc} & & \frac{dF}{dt}=\lambda_{F}+\lambda_{2}L-\mu_{F}F, \end{array}$$ (7)

$$\begin{array}{ccc} & & \frac{dB}{dt}=\lambda_{B}+\lambda_{3}F+\frac{k_{5}k_{4}^{2}}{k_{4}^{2}+\delta T^{2}}-\mu_{B}B, \end{array}$$ (8)

$$\begin{array}{ccc} & & \frac{dX}{dt}=\lambda_{X}+\frac{k_{7}k_{6}^{2}}{k_{6}^{2}+\gamma B^{2}}-\mu_{X}X. \end{array}$$ (9)

**Table 1:** Parameters in the model. P = Parameter.

P	Description	Dimensional Value	Refs.
Intracellular signaling			
λ_*F*_	Source of NF-*κ*B	3.5 × 10^−2^ *μMh*^−1^	[89]
λ_*B*_	Source of Bcl-2	8.5 × 10^−4^ *μMh*^−1^	Estimated
λ_*X*_	Source of BAX	3.3 × 10^−4^ *μMh*^−1^	[89]
λ_2_	Activation rate of NF-*κ*B by IL-6	9.15 × 10^8^ *μMmm*^3^ *g*^−1^ *h*^−1^	Estimated
λ_3_	Activation rate of Bcl-2 by NF-*κ*B	1.38 × 10^−2^ *h*^−1^	Estimated
*k*_4_	Inhibition saturation parameter	1	[89]
*δ*	Inhibition strength of Bcl-2 by TZB	1.44 × 10^18^ *mm*^6^ *g*^−2^	Estimated
*k*_5_	Autocatalytic enhancement strength of Bcl-2	1.25 × 10^−1^ *μMh*^−1^	Estimated
*k*_6_	Inhibition saturation parameter	1	[89]
*γ*	Inhibition strength of BAX by Bcl-2	8 *μM*^−2^	[89], Estimated
*k*_7_	Autocatalytic enhancement strength of BAX	1*μMh*^−1^	Estimated
*μ*_*F*_	Decay rate of NF-*κ*B	3 × 10^−1^ *h*^−1^	[89, 95, 96]
*μ*_*B*_	Decay rate of Bcl-2	3.47 × 10^−2^ *h*^−1^	[97]
*μ*_*X*_	Decay rate of BAX	2 × 10^−2^ *h*^−1^	[89, 95, 96, 98]
Production rates			
*r*	Growth rate of cancer	7 × 10^−2^ *h*^−1^	[52, 89, 99, 100], Estimated
*K*	Carrying capacity of cancer cells	2.5 × 10^−3^ *gmm*^−3^	[3, 4, 52, 89], Estimated
λ_1_	Production rate of cancer cells by CSC	5 × 10^−14^ *h*^−1^	Estimated
*β*	Production rate of CSC by TZB	3.12 × 10^−17^ *h*^−1^	Estimated
λ_*L*_	Supply rate of IL-6	1.34 × 10^−11^ *gmm*^−3^ *h*^−1^	Estimated
*k*_3_	Signaling strength of IL-6 by CSC	6.56 × 10^−17^ *h*^−1^	Estimated
Decay rates			
*μ*_*C*_	Death rate of cancer cells by apoptosis	3 × 10^−3^ *h*^−1^	Estimated
*α*	Scaling factor in the Bcl-2-induced apoptosis	2 *μM*	Estimated
*k*_1_	Scaling parameter in inhibition of IL-6 production	1	Estimated
*k*_2_	Inhibition strength of IL-6 by ATV	5 × 10^−2^ *μM*^−1^	Estimated
*μ*_*L*_	Decay rate of IL-6	8.15 × 10^−2^ *h*^−1^	[101, 102]
*μ*_*T*_	Decay rate of TZB	5.0 × 10^−3^ *h*^−1^	[103]
*μ*_*A*_	Decay rate of ATV	4.95 × 10^−2^ *h*^−1^	[104]
Therapeutic			
*I*_*A*_	ATV injection dose	0–800 *μMh*^−1^	[10, 105], Estimated
*I*_*T*_	TZB injection dose	(0–3.75) × 10^−10^ *gmm*^−3^ *h*^−1^	[7, 10], Estimated

Note that the up-regulation of BAX and down-regulation of Bcl-2 induce apoptosis while the down-regulation of BAX and up-regulation of Bcl-2 result in anti-apoptosis status. Therefore, the system select either TZB-mediated apoptosis or IL-6-induced anti-apoptosis of cancer cells based on up- or down-regulation of these signaling networks (NF*κ*B, Bcl-2, BAX).

Stability analysis of the intracellular system (7)–(9) is given in S1 Text.

### Cancer cell density (=*C*(*t*))

Several growth models such as Gompertz, logistic, and nonlinear growth curves have been used to fit to the empirical data on tumor growth curve [51]. In particular, logistic models have successfully shown to predict these experimental results [3, 4, 46, 52–61] in the presence and absence of growth factors. CSCs play an important role in progression and metastasis of cancer cells by providing potential cells and regulating intermediate steps [62]. We assume that (i) cancer cells grow at a basic rate *r* with a carrying capacity *K*, following logistic growth, (ii) cancer stem cells provide a source of the cancer cell component at a rate λ_1_, and (iii) cancer cells are eliminated from the system in response to the TZB treatment via the apoptosis intracellular signaling network.

The mass balance of the cancer cell density leads to a differential equation as follows:

$$\begin{array}{ccc} & & \frac{dC}{dt}=rC(1-\frac{C}{K})+\lambda_{1}S-\mu_{C}\frac{\alpha }{B}CI_{apop}, \end{array}$$ (10)

where *μ*_*C*_ is the cancer cell killing rate by the apoptosis process. Here *I*_*apop*_ is an indicator function of apoptosis which can be either one or zero depending the level of Bcl-2 (*B*) and BAX (*X*):

$$\begin{array}{c} I_{apop}=\{\begin{array}{cc} 1 & \text{if}\;B<th_{B},\;X>th_{X}\\ 0 & \text{otherwise}, \end{array} \end{array}$$ (11)

where *th*_*B*_ and *th*_*X*_ are the threshold values of Bcl-2 and BAX, respectively. Strong Bcl2 expression reduces the strength of cellular apoptosis, i.e. the killing rate of cancer cells is reversely proportional to the Bcl-2 level. See the fraction form, $\frac{\alpha }{B}$, in the third term of Eq (10) where *α* is a scaling factor.

### Cancer stem cell density (=*S*(*t*))

Trastuzumab was shown to induce an increase in CSCs as well as the acquisition of resistance [63]. For example, long term treatment of TZB stimulates highly enriched CSCs environment which presents an EMT phenotype secreting huge amount of IL-6 (>100-fold) relative to parental type [13]. We assume that CSCs are activated after long term treatment of TZB (20 days) and the activation is sustained for 5 days. The governing equation of the CSC density is

$$\begin{array}{ccc} & & \frac{dS}{dt}=\beta TI_{s}, \end{array}$$ (12)

where *β* is the production rate of CSC by TZB and *T* is the TZB concentration. Here, *I*_*s*_ is an indicator function that activates and deactivates the CSC production in response to TZB treatment:

$$\begin{array}{c} I_{s}=\{\begin{array}{cc} 1 & \text{if}\;20\;\text{days}\;<t\leq 25\;\text{days},\\ 0 & \text{otherwise}. \end{array} \end{array}$$ (13)

### Concentration of IL-6 (=*L*(*t*))

Activated CSCs from long term TZB treatment were shown to secrete a high level of IL-6 (>100-fold) [13]. On the other hand, ATV treatment was shown to rescue the TZB-mediated CRCC [10]. In particular, the up-regulated IL-6 expression from TZB treatment can be significantly reduced in this ATV-TZB combination therapy [10]. We assume that IL-6 is highly secreted from CSCs but ATV inhibits its supply. Then, the governing equation for IL-6 is

$$\begin{array}{ccc} & & \frac{dL}{dt}=\lambda_{L}+\frac{k_{3}S}{k_{1}+k_{2}A}-\mu_{L}L, \end{array}$$ (14)

where λ_*L*_ is the basic supply rate of IL-6 from blood vessels, *k*_3_ represents the secretion rate of IL-6 from CSCs, *k*_1_ is the scaling parameter, *k*_2_ is the inhibition strength of IL-6 by ATV, and *μ*_*L*_ is the decay rate of IL-6.

The model parameters is provided in Table 1. Parameter estimation and nondimensionalization of the system (7)–(14) are given in S2 Text. All simulations of the mathematical model were performed using a computation software Matlab (www.mathworks.com) for ordinary differential equations (4th order nonlinear ode solver *ode45*).

## Results

### Dynamics of intracellular system

**Fig 3:**
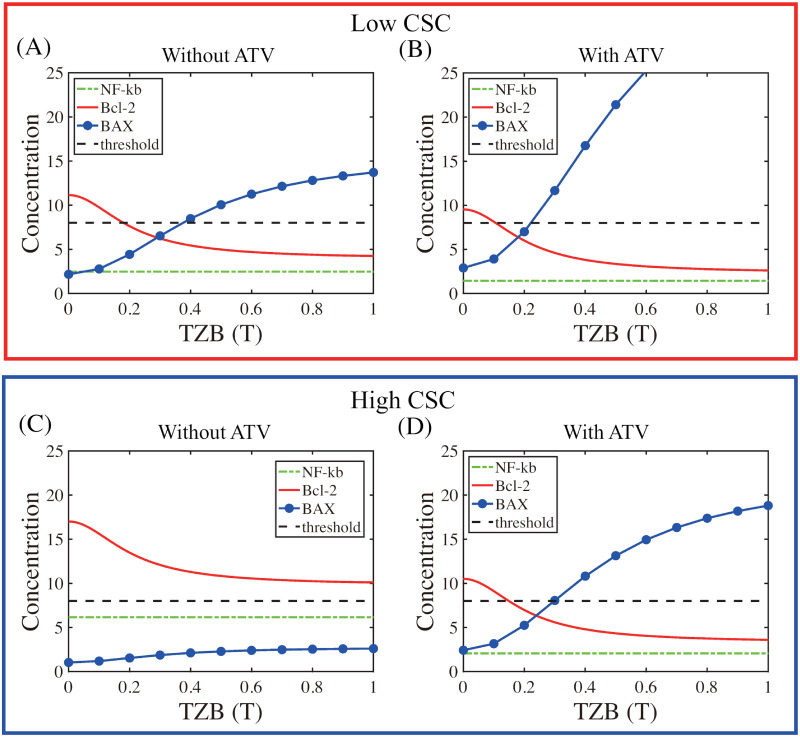
The effects of TZB on intracellular variables in response to low (A-B) and high (C-D) CSC density. (A,B) Steady states of concentrations of NF*κ*B, Bcl-2, and BAX in response to TZB treatment in the absence (A) and presence (B) of ATV when the CSC density is low. (C,D) Responses of intracellular variables without (C) and with (D) ATV treatment when the CSC density is high. *threshold value = dotted line.

We investigated the local dynamics of the intracellular system (7)–(9) in response to various CSC conditions. We recall that apoptosis is induced by down-regulation of Bcl-2 and up-regulation of BAX while anti-apoptosis is induced by up-regulation of Bcl-2 and down-regulation of BAX. When the main IL-6-NF*κ*B-Bcl-2-BAX system (7)–(9),(14) is in equilibrium, we can solve levels of NF*κ*B (*F*), Bcl-2 (*B*), and BAX (*X*) as a function of injected TZB amounts (*T*; 0 ≤ *T* ≤ 1) for any set of parameters λ_*F*_, λ_*B*_, λ_*X*_, λ_*L*_, λ_*i*_, *k*_*j*_, *δ*, *γ*, *μ*_*F*_, *μ*_*B*_, *μ*_*X*_, *μ*_*L*_. Fig 3 shows the graphs *F* = *F*(*T*), *B* = *B*(*T*), *X* = *X*(*T*) as curves with essential intracellular parameters in Table 1 in response to low (Fig 3A and 3B; *S* = 0.25) and high (Fig 3C and 3D; *S* = 1.0) CSC stimuli. When the CSC density is low and without ATV treatment, if *T* is small, the Bcl-2 level is high and the BAX level is low. As *T* is increased, the Bcl-2 expression is decreased due to inhibition mechanism from *T* while the BAX level is increased (> *th*, the threshold value) due to down-regulation of Bcl-2. See Fig 3A. When ATV treatment is added to the system, the BAX level shows ultrasensitivity to the TZB level (blue curve in Fig 3B) relative to the control case in Fig 3A but there are minor changes in Bcl-2 and NF*κ*B. This sharp increase in BAX expression in the adjuvant ATV therapy essentially increases the probability of switching to the apoptosis status in response to relatively lower TZB doses. For example, the system with ATV treatment will lead to apoptosis when *T* ∼ 0.25 but the system without ATV will not allow this and maintains anti-apoptosis status of cancer cells. In both cases, the NF*κ*B level is not significantly affected by this change in *T*, showing kinetic low sensitivity against TZB dose variations and independency from the smaller downstream network (TZB-Bcl-2-BAX).

Recall that long treatment of patients with TZB typically leads to formation of a CSC-rich tumor microenvironment. When the CSC density is high (Fig 3C and 3D), the general increasing and decreasing patterns of Bcl-2 and BAX, respectively, as a function of TZB are same as in the case with the low CSC density (Fig 3A and 3B). However, the IL-6 level is much higher (*L* ∼ 60), leading to the higher NF*κ*B level, in the absence of ATV treatment (Fig 3C) and is low (*L* ∼ 18), leading to the lower NF*κ*B level, in the presence of ATV treatment (Fig 3D). In the TZB single treatment case in Fig 3C, this over-expression of NF*κ*B (green dashed) results in a significant uniform increase in Bcl-2 (red solid) and heavy suppression of BAX expression (blue circle) regardless of TZB stimuli, supporting the anti-apoptosis status, relative to the case with the low CSC density (Fig 3A). In other words, TZB treatment alone is not effective in killing cancer cells in a CSC-rich microenvironment. On the other hand, a combination (TZB+ATV) therapy (Fig 3D) can increase the anti-tumor efficacy by decreasing the overall expression levels of IL-6, NF*κ*B (green dashed), and Bcl-2 (red solid), and by increasing the overall BAX levels (blue circle). Therefore, the adjuvant therapy of ATV is very effective in killing cancer cells in response to high TZB doses (*T* > 0.4).

These results clearly suggest the effect of CSCs and intracellular responses to TZB treatment in the presence and absence of ATV. By observing simulation results on these dynamical changes in the up- or down-regulation of key intracellular molecules, we can now set the threshold values of Bcl-2 and BAX (*th*_*B*_ = *th*_*X*_ = 8). Our choices on the threshold values will be validated with consistent experimental outcomes such as dynamical changes in Bcl-2 and IL-6 under various conditions [10] later. We now define the anti-apoptotic ($T_{t}$) and apoptotic ($T_{a}$) regions as follows:

$$\begin{array}{ccc} & & T_{t}=\left\{\left(X,B\right)\in R^{2}:X<th_{X},B>th_{B}\right\}, \end{array}$$ (15)

$$\begin{array}{ccc} & & T_{a}=\left\{\left(X,B\right)\in R^{2}:X>th_{X},B<th_{B}\right\}. \end{array}$$ (16)

**Fig 4:**
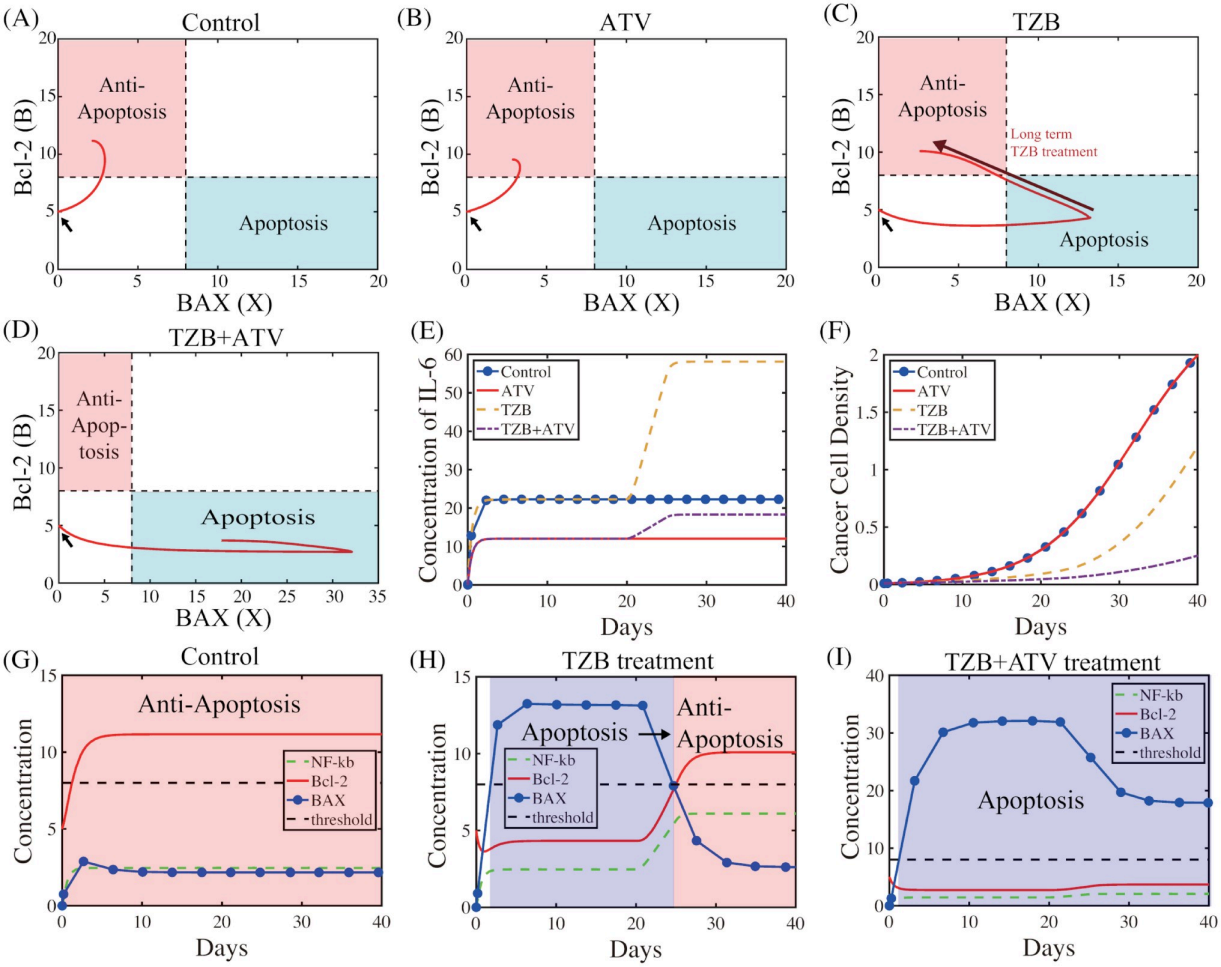
The role of TZB and ATV in regulation of the apoptosis control. (A-D) Trajectories of intracellular solutions (*X*(*t*), *B*(*t*)) in the *X*-*B* plane in response to control (TZB^−^ATV^−^), ATV alone (TZB^−^ATV^+^), TZB alone (TZB^+^ATV^−^), and combination therapy (TZB^+^ATV^+^). Initial condition (marked in black arrow): (*L*(0), *N*(0), *B*(0), *X*(0), *S*(0), *C*(0)) = (0,0,5,0,0.25,0.01). Red arrow = transition of solutions from $T_{a}$- to $T_{t}$-mode after long treatment of TZB. (E,F) Time course of the IL-6 concentration (E) and tumor density (F) in response to four cases in (A-D). (G-I) Time courses of concentrations of three intracellular variables (NF*κ*B, Bcl-2, BAX) for control (G), TZB alone (H), and combination therapy (I), respectively.

A tumor responds to initial TZB treatment but long treatment of TZB can lead to an increase in the CSC population later time, leading to resistance to TZB treatment [10]. In Fig 4, we show the temporal dynamics of concentrations of key intracellular variables (NF*κ*B, BAX, Bcl-2), IL-6, and CSCs as well as phase plane dynamics in the *X*-*B* plane in response to control (*T* = 0, *A* = 0; TZB^−^ATV^−^), ATV alone (*T* = 0, *A* = 5; TZB^−^ATV^+^), TZB alone (*T* = 1.0, *A* = 0; TZB^+^ATV^−^) and combination treatment (*T* = 1.0, *A* = 5; TZB^+^ATV^+^). The full system (7)–(14) were solved with an initial condition (*L*(0), *N*(0), *B*(0), *X*(0), *S*(0), *C*(0)) = (0, 0, 5, 0, 0.25, 0.01). The anti-apoptotic ($T_{t}$) and apoptotic ($T_{a}$) regions in Eqs (15) and (16) were marked in pink and blue boxes, respectively. Starting with an initial condition with a neutral state (marked in black arrows), the system in the absence of ATV and TZB (control) converges to the $T_{t}$-region (Fig 4A), leading to active tumor cell proliferation. In the presence of ATV treatment alone, up-regulation of Bcl-2 and down-regulation of BAX are same as the control case (Fig 4G) and the solution (*X*(*t*), *B*(*t*)) converges to the same anti-apoptosis region (Fig 4B). On the other hand, TZB treatment alone can initially lead the system to the $T_{a}$-mode (blue box in Fig 4C; blue box in Fig 4H), activating active tumor cell killing (yellow dashed in Fig 4F) compared to control or ATV^+^ case. However, formation of CSCs in TME after *t* = 20 *days* leads to up-regulation of IL-6 (yellow dashed in Fig 4E) and sharp transition from apoptotic to anti-apoptotic status (black arrow in Fig 4H; blue→pink box) by increasing Bcl-2 (red solid in Fig 4H) from increased NF*κ*B level (green dashed in Fig 4H) and suppression of BAX (blue circle in Fig 4H). This high level of IL-6 can cause CRCC, the serious side effect of TZB treatment. In the combination (TZB^+^ATV^+^) therapy, ATV can effectively suppress IL-6 expression (dot-dashed in Fig 4E), which can down-regulate the levels of NF*κ*B (green dashed in Fig 4I) and Bcl-2 (red solid in Fig 4I). Then, the up-regulation of BAX (*X* > *th*_*X*_; blue circle in Fig 4I) leads to apoptosis of cancer cells (Fig 4D) even in the presence of CSCs in TME and induces synergistic effect of cancer cell killing (dot-dashed in Fig 4F). More importantly, the combination treatment can reduce or negate the side effect of long term TZB injection, CRCC, in addition to high anti-tumor efficacy.

### Effect of TZB on tumor growth

**Fig 5:**
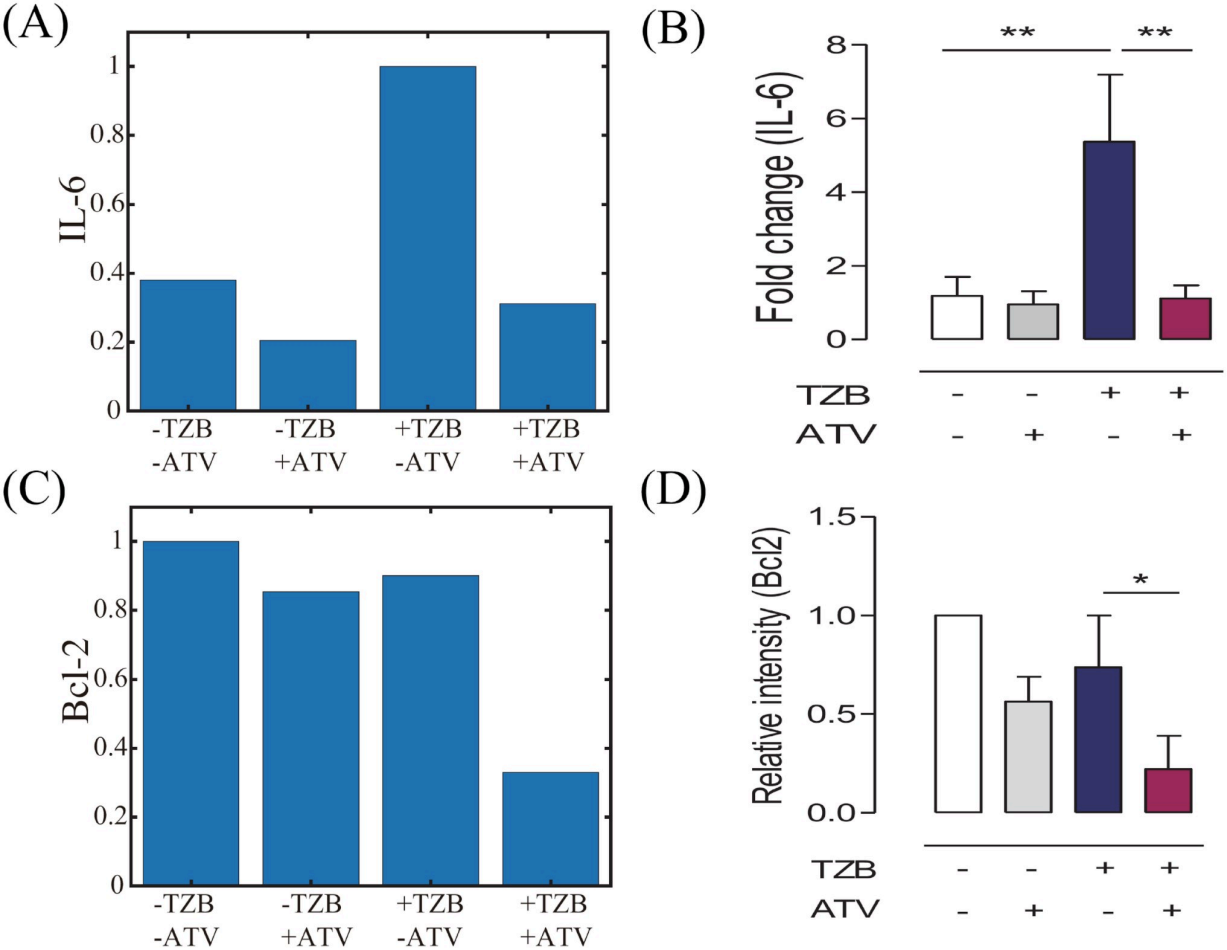
Comparison with experimental results. (A,C) IL-6 levels (A) and Bcl-2 expression (C) at final time (*t* = 30 *days*) for control (TZB^−^ATV^−^), ATV alone (TZB^−^ATV^+^), TZB alone (TZB^+^ATV^−^), and combination therapy (TZB^+^ATV^+^). (B,D) IL-6 levels (B) and Bcl-2 expression (D) were measured in experimental behavioral study [10] where the passive avoidance test was adapted for quantitative assessment of the chemical response to an aversive shock and long-term memory (figures from [10] with permission).

Fig 5A illustrates the biochemical effect of on IL-6 expression in response to four test frames as before: control (TZB^−^ATV^−^), ATV alone (TZB^−^ATV^+^), TZB alone (TZB^+^ATV^−^), and combination therapy (TZB^+^ATV^+^). The IL-6 level is significantly decreased (∼46% reduction) in the ATV treatment case (TZB^−^ATV^+^) relative to the control case, illustrating the strong suppression of IL-6 expression by ATV doses. On the other hand, the combination therapy can reduce the IL-6 expression by 69% relative to the TZB treatment (TZB^+^ATV^−^), showing strong neutralizing effect of ATV on CRCC in the TZB-driven anti-cancer therapy. Our phenomenological model is able to qualitatively capture experimental observations (Fig 5B), significant increase in the IL-6 levels in TZB treatment and the negating effect of ATV in the combination therapy, in a passive avoidance test [10] where biochemical changes in IL-6 levels in response electric stimulation were measured in four different test beds. Fig 5C shows Bcl-2 expression in the corresponding four comparison groups. The significant down-regulation of the Bcl-2 level in response to a combination therapy (Fig 5C) eventually leads to the apoptotic status and efficient tumor cell killing as well as prevention of CRCC (Fig 5A). Therefore, our phenomenological model is able to reproduce these responses from experimental data (Fig 5D) [10] in four cases.

### Effect of ATV on cognitional activity

**Fig 6:**
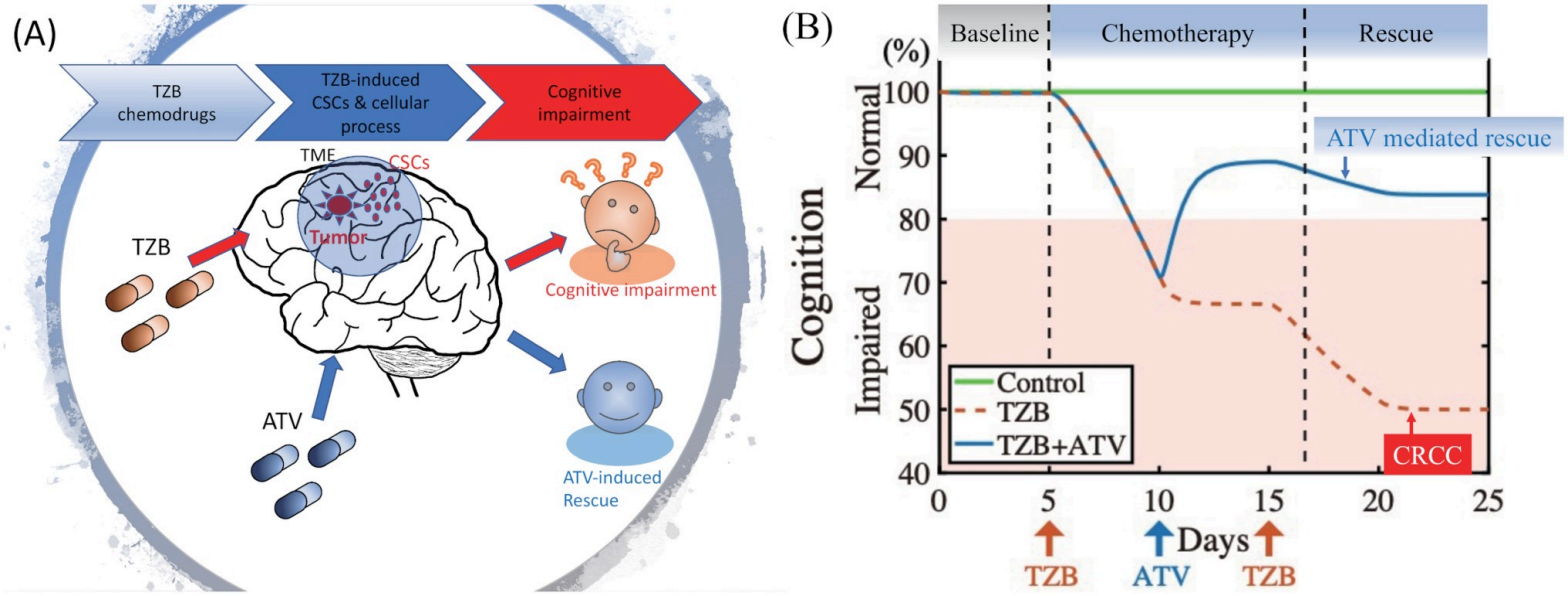
Effect of existing approved drugs, ATV, on cognitional activity in a combination cancer therapy. (A) Even though the current clinical therapy is to prescribe chemodrugs such as TZB to treat the growing tumor in tumor microenvironment (TME), a more aggressive, targeted combination approach is to prescribe existing other drugs, such as ATV, that improves the cognition level and normalize likely convergent cellular networks. (B) With the combination therapy (TZB+ATV), we can aim to directly improve the temporal trajectory of CRCC and to suppress tumor growth, through suppression of IL-6 and recovering apoptosis of cancer cells after chemotherapy, returning cognitive capability to the normal level.

Considering the complexity of applying new therapeutic compounds for the CNS [64, 65], we propose that prescription of existing drugs such as ATV can be a feasible approach to successfully treating CRCC during TZB treatment (Fig 6A). In spite of the heterogeneity of molecular mechanisms, there exist convergent cellular mechanisms as a target [66]. Even though the current clinical therapy is to prescribe chemodrugs such as TZB to treat the growing tumor, necessarily resulting in cognitive impairment (red dotted curve in Fig 6B), a more aggressive application of ATV can improve the cognition level and normalize convergent cellular networks (blue solid curve in Fig 6B). With this combination therapy (TZB+ATV), we can aim to directly improve the temporal trajectory of CRCC and to suppress tumor growth, through suppression of IL-6 and recovering apoptosis of cancer cells after chemotherapy, returning cognitive capability to the normal level (Fig 6B).

**Fig 7:**
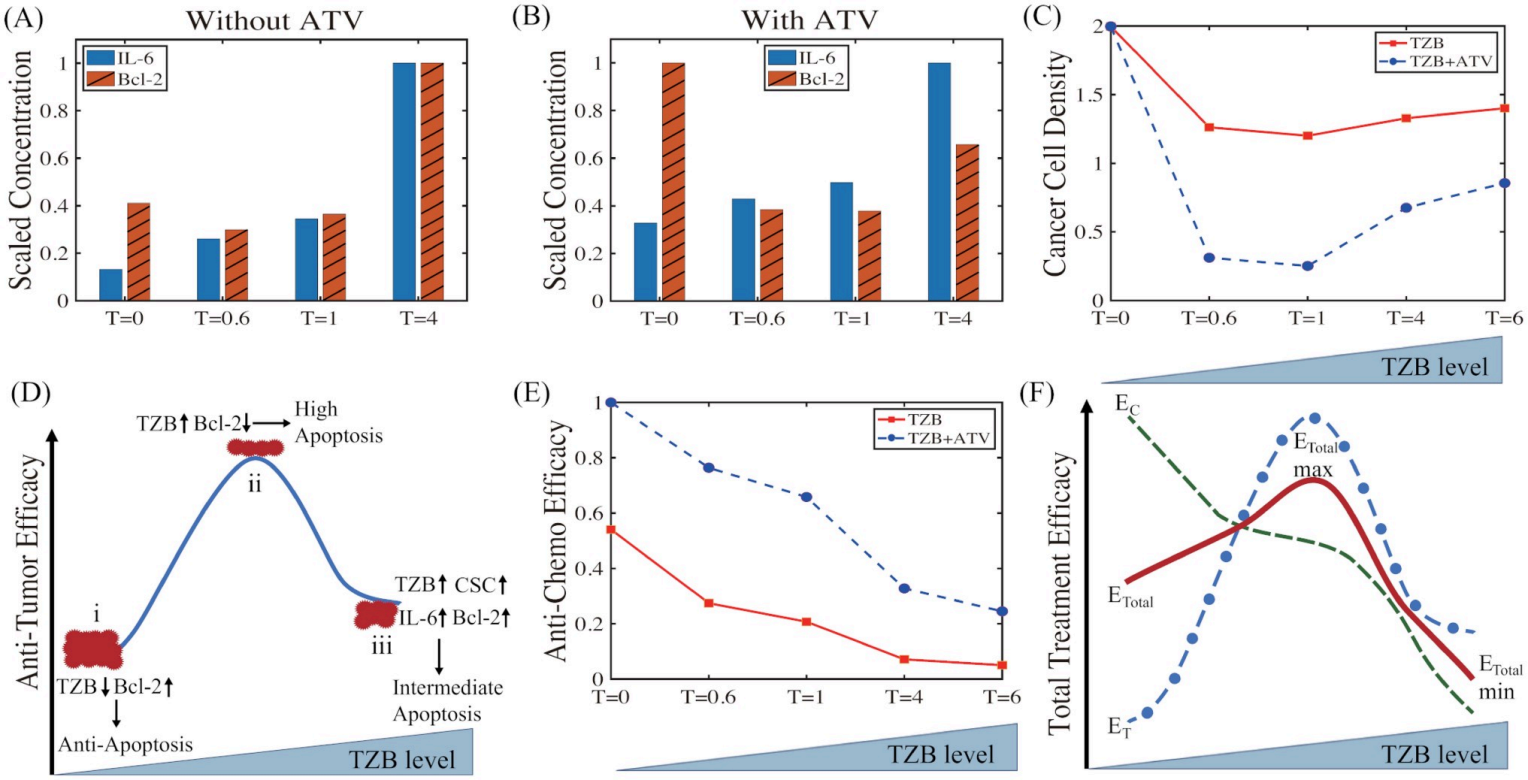
Nonlinear dynamics of the system in response to TZB treatment. (A,B) IL-6 and Bcl-2 levels at 30 days post treatments in response to various TZB doses (*T* = 0, 0.6, 1.0, 4.0) in the absence (A) and presence (B) of ATV adjuvant therapy. *The values in (A) were normalized against the case with *T* = 4, and the IL-6 and Bcl-2 values in (B) were normalized against *T* = 4 and *T* = 0, respectively. (C) Cancer cell density corresponding to (A,B). (D) A model depicting the impact of TZB and ATV combination therapy on anti-tumor efficacy (*E*_*T*_) as a function of TZB doses. (E) Anti-chemo efficacy (*E*_*c*_) with single (solid red square; TZB) and combination therapy (blue dotted circle; TZB+ATV) for various TZB doses (*T* = 0, 0.6, 1.0, 4.0, 6.0). (F) Schematic of total treatment efficacy (*E*_*Total*_; red solid curve) of the combination therapy based on *E*_*T*_ (green dashed) and *E*_*c*_ (blue circle): *E*_*Total*_ = *ω*_1_*E*_*T*_ + *ω*_2_*E*_*c*_ with *ω*_1_ = *ω*_2_ = 0.5.*ATV level in (B-F) is fixed (*A* = 5.0). Other parameters are given in Table 1.

We investigated the effect of single and combination therapy on anti-tumor efficacy and side effects, CRCC, in response to various TZB doses. Fig 7A and 7B shows the normalized activities of IL-6 (blue bar) and Bcl-2 (red shaded bar) in the single (Fig 7A) and combination (Fig 7B) therapy at 30 days for various TZB doses (*T* = 0, 0.6, 1.0, 4.0). In the absence of ATV (Fig 7A), IL-6 activities are increased as the TZB dose is increased, increasing the chance of occurrence of the CRCC phenomenon. While the IL-6 level is also increased in response to higher TZB doses in the presence of ATV (*A* = 5.0) (Fig 7B), the absolute value of IL-6 without ATV for each TZB dose is much higher than the corresponding case with ATV, illustrating chemo-suppressive effect of ATV (*cf*. Fig 5). Dose response of TZB on Bcl-2 activity at 30 days post treatments (single therapy Fig 7A; combination therapy in Fig 7B) revealed a nonlinear, V-shaped curve, indicating a dose-dependent decrease in Bcl-2 activation until a threshold, after which there was an increase. When the TZB dose is too high in the absence of ATV, emergence of CSCs in TME after 20 days leads to an increase in IL-6 expression, resulting in up-regulation of Bcl-2 and less efficient cancer cell killing (red solid square in Fig 7C) than the intermediate TZB case through the nonlinear feedback in the TZB-CSC-IL-6-NF*κ*B-Bcl-2 circuit (Fig 1). This nonlinear dose response is enhanced even further in the combination therapy. Initially, Bcl-2 activities are significantly reduced by the combination therapy as the TZB dose is increased (*T* = 0 → *T* = 1.0 in Fig 7B), leading to efficient cancer cell killing (*T* = 0 → *T* = 1.0 in Fig 7C). However, the anti-apoptotic activity by Bcl-2 is rescued when the TZB dose is increased further (*T* = 1.0 → *T* = 4.0 in Fig 7B) while IL-6 activity is still increased (blue bar; Fig 7B), which prevents efficient cancer cell killing (*T* = 1.0 → *T* = 6.0 in Fig 7C).

Bcl-2 is a key anti-apoptosis pathway of cancer cells and the nonlinear V-shaped dose response led to a bell-shaped curve of anti-tumor efficacy (Fig 7D). Here, the anti-tumor efficacy *E*_*T*_(*t*) is defined to be the reciprocal for cancer cell density at time *t*, *i.e*., $E_{T}\left(t\right)=\frac{f_{T}}{C(t)}$ where *f*_*T*_ is a scale factor. The anti-tumor efficacy is inversely proportional to the cancer cell population, *C*(*t*), at a given time *t*. Therefore, our expression, constant/*C*(*t*), represents a degree of anti-tumor efficacy among other metrics. While the intermediate level of TZB treatment attains the maximal *E*_*T*_ due to up-regulated Bcl-2 expression and sustained apoptosis activity (the case (ii)), high doses of TZB leads to lower anti-tumor efficacy from the large population of CSCs (the case (iii)). For the case of anti-CRCC efficacy, the IL-6 level can be used as a prognostic factor of CRCC and we use a similar expression for representation of the degree of anti-CRCC efficacy, *i.e*., we define the anti-CRCC efficacy *E*_*c*_(*t*) to be the reciprocal for the IL-6 level at time *t*, *i.e*., $E_{c}\left(t\right)=\frac{f_{c}}{L(t)}$ where *f*_*c*_ is a scale factor. Fig 7E shows *E*_*c*_ of single and combination therapies as a function of the TZB dose (*T* = 0.0, 0.6, 1.0, 4.0, 6.0). Here, we take *f*_*c*_ to be the maximum value *E*_*c*_ of the combination therapy when *T* = 0. In both cases, the anti-chemo efficacy monotonically decreases as the TZB dose increases while the degree of anti-chemo efficacy in the combination therapy (blue circles in Fig 7E) is uniformly larger than the single therapy case (red squares in Fig 7E). We can measure the total treatment efficacy (*E*_*Total*_) of a combination therapy by combining *E*_*T*_ and *E*_*c*_: *E*_*Total*_ = *ω*_1_*E*_*T*_ + *ω*_2_*E*_*c*_ where *ω*_1_, *ω*_2_ (*ω*_1_, *ω*_2_ ∈ [0, 1], *ω*_1_ + *ω*_2_ = 1) are the weight of *E*_*T*_ and *E*_*c*_, respectively. Fig 7F illustrates total treatment efficacy of the TZB-ATV combination therapy *E*_*Total*_ with *ω*_1_ = *ω*_2_ = 0.5 as a function of the TZB dose. The total treatment efficacy attains its maximum value at the intermediate level of TZB (*cf*. Fig 7D). However, the system reaches its minimum value of *E*_*Total*_ at the highest TZB dose. Therefore, high doses of TZB treatment are not a favorable option in order to maximize anti-tumor efficacy and minimize CRCC. The model predicts that best clinical outcomes can be achieved from the combination therapy with intermediate doses of TZB.

### Therapeutic strategies by ATV injection

We now investigate the effect of ATV infusion administration on tumor growth. We assume that ATV is injected over time intervals [*t*_*i*_, *t*_*i*_ + *h*_*A*_], i = 1,…,*N*_*A*_ with the time duration *h*_*A*_, and a period *τ*_*A*_ (=*t*_*i*+1_ − *t*_*i*_, *i* = 1,…, *N*_*A*_-1). Here, *N*_*A*_ is the total number of ATV injections. For simulations, we introduce the following governing equations of the ATV concentration (*A*(*t*)) in addition to Eqs (7)–(14):

$$\begin{array}{ccc} & & \frac{dA}{dt}=\sum_{i=1}^{N_{A}}I_{A}J_{\left[t_{i},t_{i}+h_{A}\right]}-\mu_{A}A \end{array}$$ (17)

where *I*_*A*_ is the ATV dose and *μ*_*A*_ is the decay rate of ATV. Here, $J_{\left[t_{i},t_{i}+h_{A}\right]}$ is an indicator function, giving 1 when *t* ∈ [*t*_*i*_, *t*_*i*_ + *h*_*A*_] or 0 otherwise.

**Fig 8:**
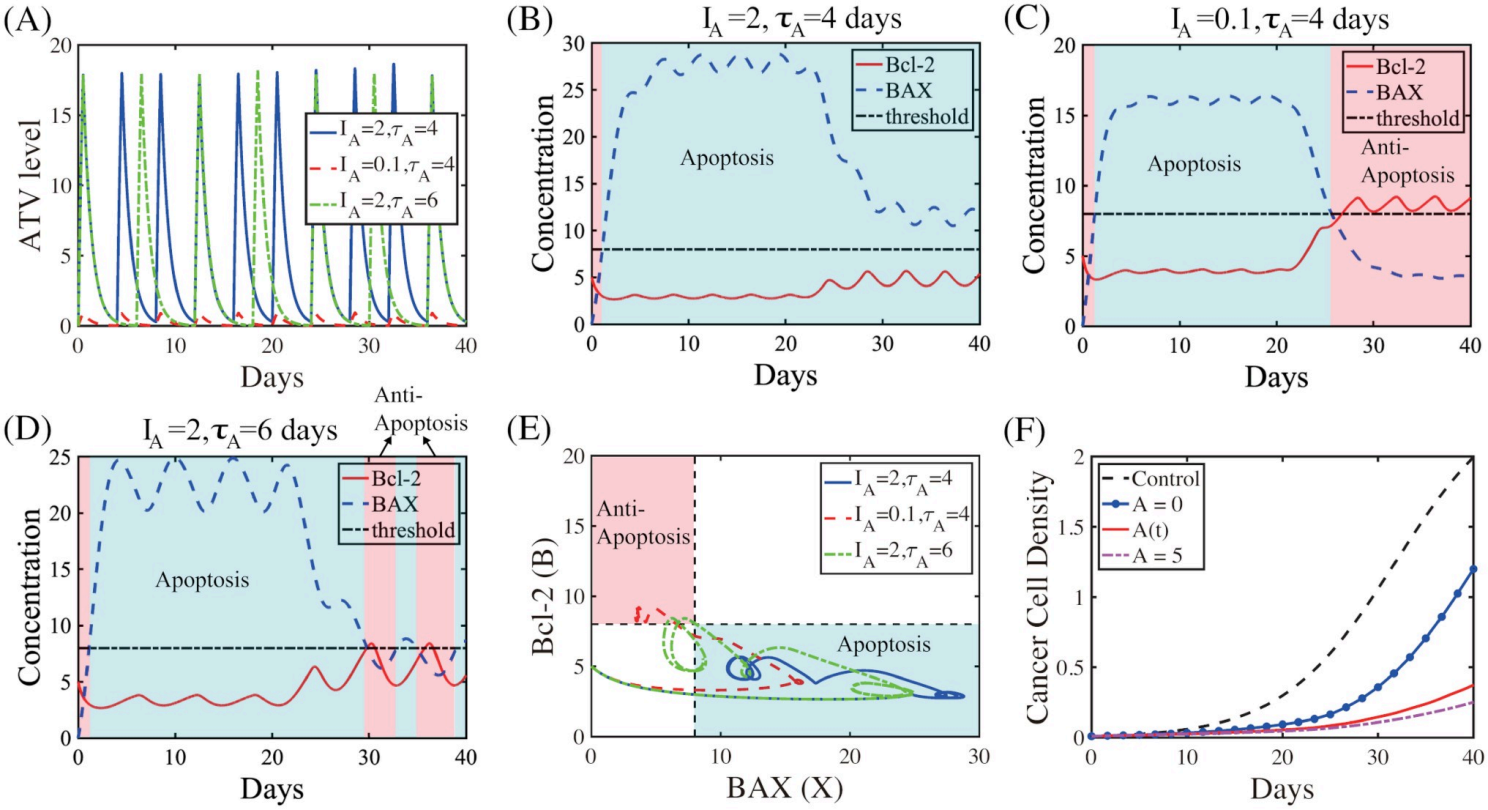
Anti-tumor efficacy and intracellular dynamics in response to ATV infusion. (A) Time courses of the ATV levels in response to fluctuating ATV infusion with various doses (*I*_*A*_) and periods (*τ*_*A*_; days): blue solid (*I*_*A*_ = 2, *τ*_*A*_ = 4), red dashed (*I*_*A*_ = 0.1, *τ*_*A*_ = 4), green dotted (*I*_*A*_ = 2, *τ*_*A*_ = 6). (B,C,D) Time courses of the concentrations of Bcl-2 (red solid) and BAX (blue dashed) in response to fluctuating ATVs: *I*_*A*_ = 2, *τ*_*A*_ = 4 (B), *I*_*A*_ = 0.1, *τ*_*A*_ = 4 (C), *I*_*A*_ = 2, *τ*_*A*_ = 6 (D). Black dotted line = the threshold of Bcl-2 and BAX level. Apoptotic and anti-apoptotic areas are marked in light blue and pink boxes, respectively. (E) Dynamics of Bcl-2 and BAX corresponding to (B-D) in the *X* − *B* plane. (F) Time courses of the tumor density for control (black dashed; TZB^−^ATV^−^), TZB treatment alone (blue circle; *A* = 0; TZB^+^ATV^−^), periodic ATV infusion (red solid; TZB^+^ATV^+/*^) and combination treatment (pink dashed; *A* = 5; TZB^+^ATV^+^).

Fluctuating ATV levels (Fig 8A) in response to a periodic infusion of ATV with a dose (*I*_*A*_ = 2) and period (*τ*_*A*_ = 4 *days*) lead to down-regulation of Bcl-2 and up-regulation of BAX for most of time, leading to persistent apoptosis (blue box in Fig 8B), despite small-scale fluctuations. When the ATV dose is lowered (*I*_*A*_ = 2 → 0.1), the apoptotic status at earlier time (blue box in Fig 8C) transits to the anti-apoptotic status at later time (pink box in Fig 8C; *t* > 25 *days*) and the effect of TZB-ATV combination therapy is significantly reduced. When the injection period is increased (*τ*_*A*_ = 4 → 6), the fluctuation of Bcl-2 and BAX activities is increased and the apoptotic status at earlier time (blue box in Fig 8D) does not persist for a long time since relatively low ATV cannot overcome the resistance from the basal TZB therapy. This leads to the alternating transitions between $T_{a}$-mode (blue box in Fig 8D) and $T_{t}$-mode (pink box in Fig 8D) status at later times (*t* > 30 *days*). The trajectories of the intracellular solutions (*X*(*t*),*B*(*t*)) in Fig 8E corresponding to three cases illustrates specific dynamic flow of Bcl-2 and BAX in the anti-apoptotic (pink) or apoptotic (blue) regions. The effective ATV infusion with a schedule in Fig 8B (*I*_*A*_ = 2, *τ*_*A*_ = 4) leads to slower tumor growth compared to control (black dashed; TZB^−^ATV^−^ in Fig 8F) and single TZB treatment (blue circle; *A* = 0; TZB^+^ATV^−^ in Fig 8F). However, this anti-tumor efficacy from periodic ATV infusion is lower than a constant ATV infusion therapy (pink dashed; *A* = 5; TZB^+^ATV^+^ in Fig 8F).

**Fig 9:**
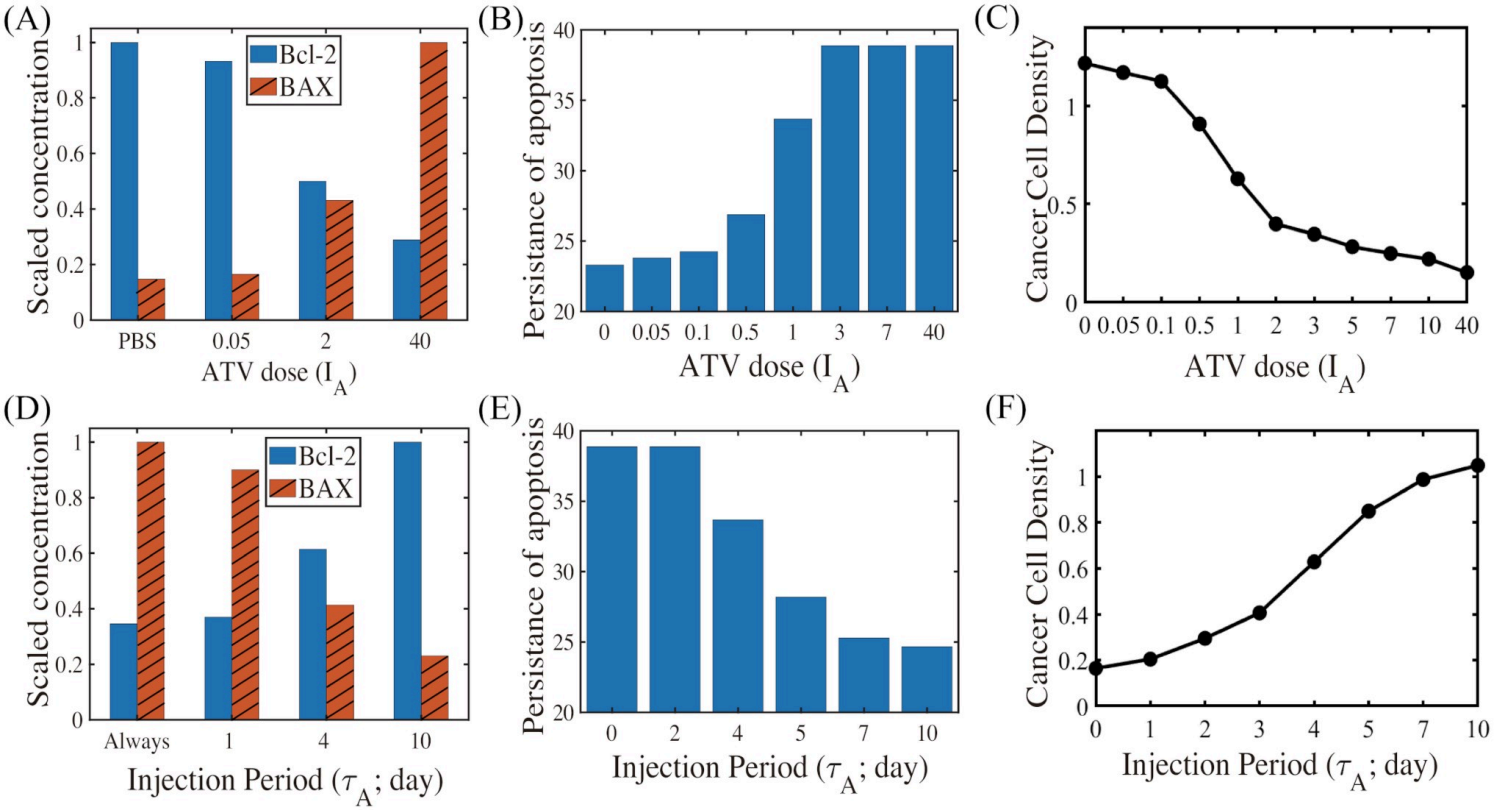
Effect of ATV injection schedule on apoptosis and tumor growth in the combination (TZB+ATV) therapy. (A) Normalized levels of Bcl-2 (blue) and BAX (red) in response to periodic ATV infusion schedule with various doses (*I*_*A*_ = 0(*PBS*), 0.05, 2, 40). (B) Persisting time of ATV-induced apoptosis status in response to various ATV doses (*I*_*A*_ = 0, 0.05, 0.1, 0.5, 1, 3, 7, 40). (C) Dose response: cancer cell density in response to various ATV doses (*I*_*A*_ = 0, 0.05, 0.1, 0.5, 1, 2, 3, 5, 7, 10, 40). *τ_*A*_ was fixed in (A-C): *τ*_*A*_ = 4 *days*. (D) Normalized levels of Bcl-2 (blue) and BAX (red) in response to periodic ATV infusion schedule with various ATV injection periods (*τ*_*A*_ = 0, 1, 4, 10 *days*). (E) Persisting time of ATV-induced apoptosis status in response to various ATV injection periods (*τ*_*A*_ = 0, 2, 4, 5, 7, 10 *days*). (F) Dose response: cancer cell density in response to various ATV injection periods (*τ*_*A*_ = 0, 1, 2, 3, 4, 5, 7, 10). *ATV dose (*I*_*A*_) was fixed in (D-F): *I*_*A*_ = 2.

In Fig 9, we investigate anti-tumor efficacy in response to the combination (TZB+ATV) therapy with various ATV infusion schedules (dose *I*_*A*_; injection period (*τ*_*A*_). As the ATV dose is increased (*I*_*A*_ = 0 → 0.05 → 2 → 40), activities of gate keeper (Bcl-2) is decreased but the apoptosis inducer (BAX) enhances its activity (Fig 9A). Naturally, the ATV dose increases the probability of persisting the apoptotic status (Fig 9B) and decreases the cancer cell population at final time. Dose response curve is shown in Fig 9C. On the other hand, as the ATV injection period is increased (*τ*_*A*_ = 0 → 1 → 4 → 10), the Bcl-2 level is increased and BAX activities are reduced (Fig 9D), reducing the probability of apoptotic death of cancer cells through decreases in persistence of apoptosis (Fig 9E). Dose response for various ATV periods (*τ*_*A*_ = 0, 1, 2, 3, 4, 5, 7, 10) is shown in Fig 9F.

**Fig 10:**
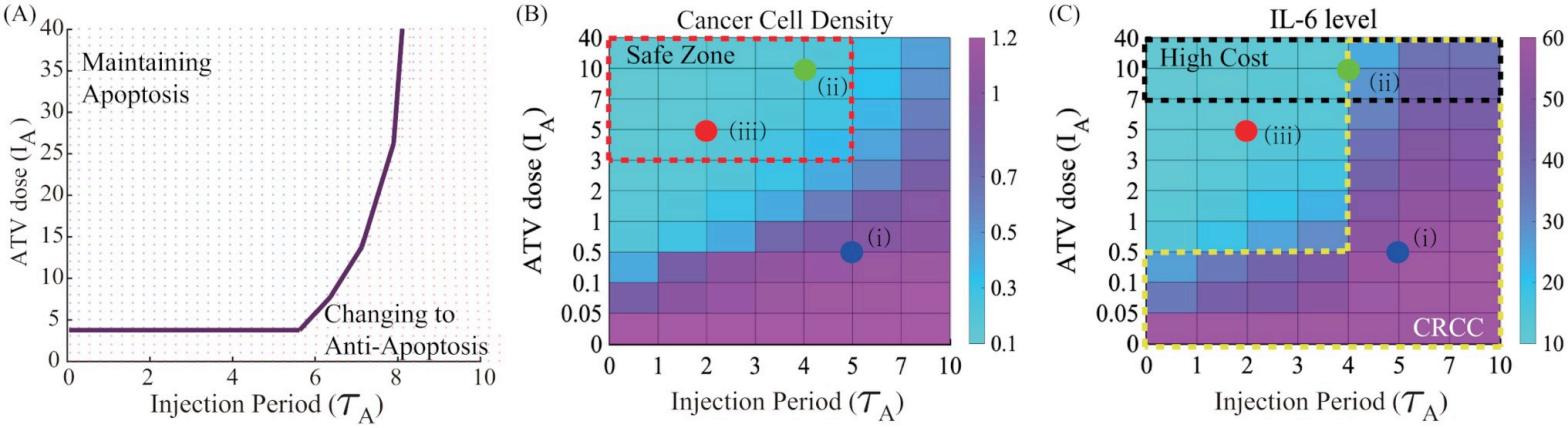
Therapeutic effect of ATV infusion in the combination (TZB+ATV) therapy. (A) Persistence of apoptosis at later times in the *τ*_*A*_ − *I*_*A*_ plane. (B,C) Cancer populations (B) and IL-6 levels in response to the combination therapy with various doses (*I*_*A*_ = 0.05, 0.1, 0.5, 1, 2, 3, 5, 7, 10, 40) and injection periods (*τ*_*A*_ = 0, 1, 2, 3, 4, 5, 7, 10) of ATV infusion at final time (*t* = 40 *days*). The best schedule ((iii); marked in red circle) can be obtained by avoiding CRCC and minimizing costs (minimizing doses and maximizing infusion periods) while keeping the low tumor size.

Recall that apoptotic status may not be maintained at later times in the combination (TZB+ATV) therapy under the conditions that ATV infusion is not strong enough (Fig 8). Fig 10A displays two regions, persistent apoptotic region (blue) vs transition to anti-apoptotic status at later times, in the *τ*_*A*_ − *I*_*A*_ plane. Fig 10B shows cancer cell populations in the combination (TZB+ATV) therapy with various doses and injection periods of ATV at final time (*t* = 40 *days*). For a fixed dose of ATV, the anti-tumor efficacy is decreased as the injection period is increased in general due to increased probability of the transition to $T_{t}$-status (Fig 10A). However, this also increases the IL-6 level (Fig 10C), putting patients at greater risk of CRCC and cognitive impairment [23–26]. For a fixed period of ATV injection, the higher ATV dose can decrease the tumor size, due to persistent $T_{a}$-status (Fig 10B), and IL-6 level (Fig 10C). Thus, a higher ATV dose can increase anti-tumor efficacy and decease probability of CRCC. However, this can also increase administrative costs at clinic and other side effects associated with the higher dose. For optimal strategy analysis, we can define the High Cost Zone ($Z_{C}$; black dotted box; Fig 10C), Safe Zone ($Z_{S}$; red dotted box; Fig 10B), CRCC Zone ($Z_{B}$; yellow dotted box; Fig 10C). For example, an injection strategy (i) belongs to $Z_{B}$ while another strategy (ii) belongs to $Z_{S}$ and $Z_{C}$. Therefore, careful infusion strategies need to be designed in order to efficiently deliver ATV to a tumor microenvironment with low costs and maximize anti-tumor efficacy while minimizing systemic side effects [23–26]. An injection strategy (iii) in the $Z_{S}$-zone can be one of the best choices by avoiding $Z_{B}$ and $Z_{C}$ zones and maximizing the injection period.

### Sensitivity analysis

In the mathematical model in this work, there are some parameters for which no experimental data are available and these may affect the simulation outcomes. We take these parameters (λ_2_, *δ*, *γ*, *r*, λ_1_, *μ*_*C*_, *k*_2_, *k*_3_) in the model for sensitivity analysis. We investigated the sensitivity of densities of cancer cells (*C*), IL-6 (*L*), NF-*κ*B (*F*), Bcl-2 (*B*) and BAX (*X*) to these parameters at several time points. We choose a range for each of these parameters of interest and divide the ranges into 10,000 subintervals of uniform length for calculation of corresponding partial rank correlation coefficient (PRCC) value. A PRCC value is a real number in the interval [−1, 1] with the sign dictating whether the given variable is increased (+) or decreased (-) in response to an increase in the parameter at a given time.

**Fig 11:**
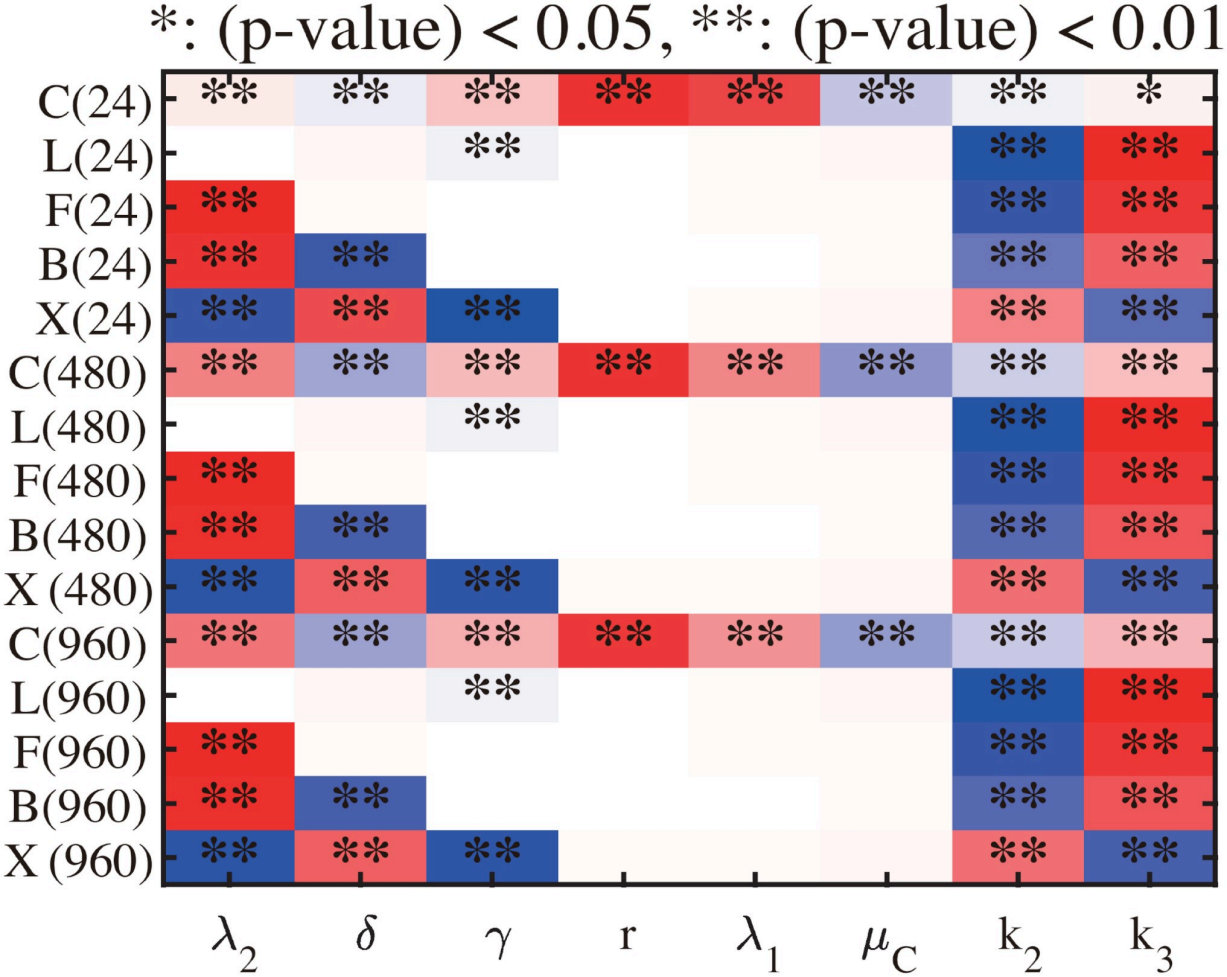
Sensitivity analysis. General Latin Hypercube Sampling (LHS) scheme and Partial Rank Correlation Coefficient (PRCC) were used for the mathematical model. The reference output in color is PRCC values (red for positive PRCC values; blue for negative PRCC values) for the density of tumor cells (*C*), concentration of IL-6 (*L*), NF-*κ*B (*F*), Bcl-2 (*B*), and BAX (*X*) at time *t* = 24, 480, 960 *h*. Analysis was carried out using the method of [67] with a sample size 10,000.

Fig 11 shows the PRCC values of all variables at *t* = 24, 480, 960 hours. The NF-*κ*B level is positively correlated to λ_2_, *k*_3_ but negatively correlated to *k*_2_. In a similar fashion, the Bcl-2 activity is positively correlated to λ_2_, *k*_3_ but negatively correlated to *δ*, *k*_2_. However, activities of apoptosis inducer, BAX, shows the opposite effect: negative correlation to λ_2_, *γ*, *k*_3_ and positive correlation to *δ*, *k*_2_. IL-6 levels are not sensitive to most parameters except *k*_2_, *k*_3_. In particular, cancer cell density has a positive correlation with *r*, λ_1_ but are negatively correlated to *μ*_*C*_ (S3 Text). The sensitivity analysis in this section was carried out using the method from [67] and Matlab files available from the website of Denise Kirschner’s Lab: http://malthus.micro.med.umich.edu/lab/usadata/.

### Optimized schedule

**Fig 12:**
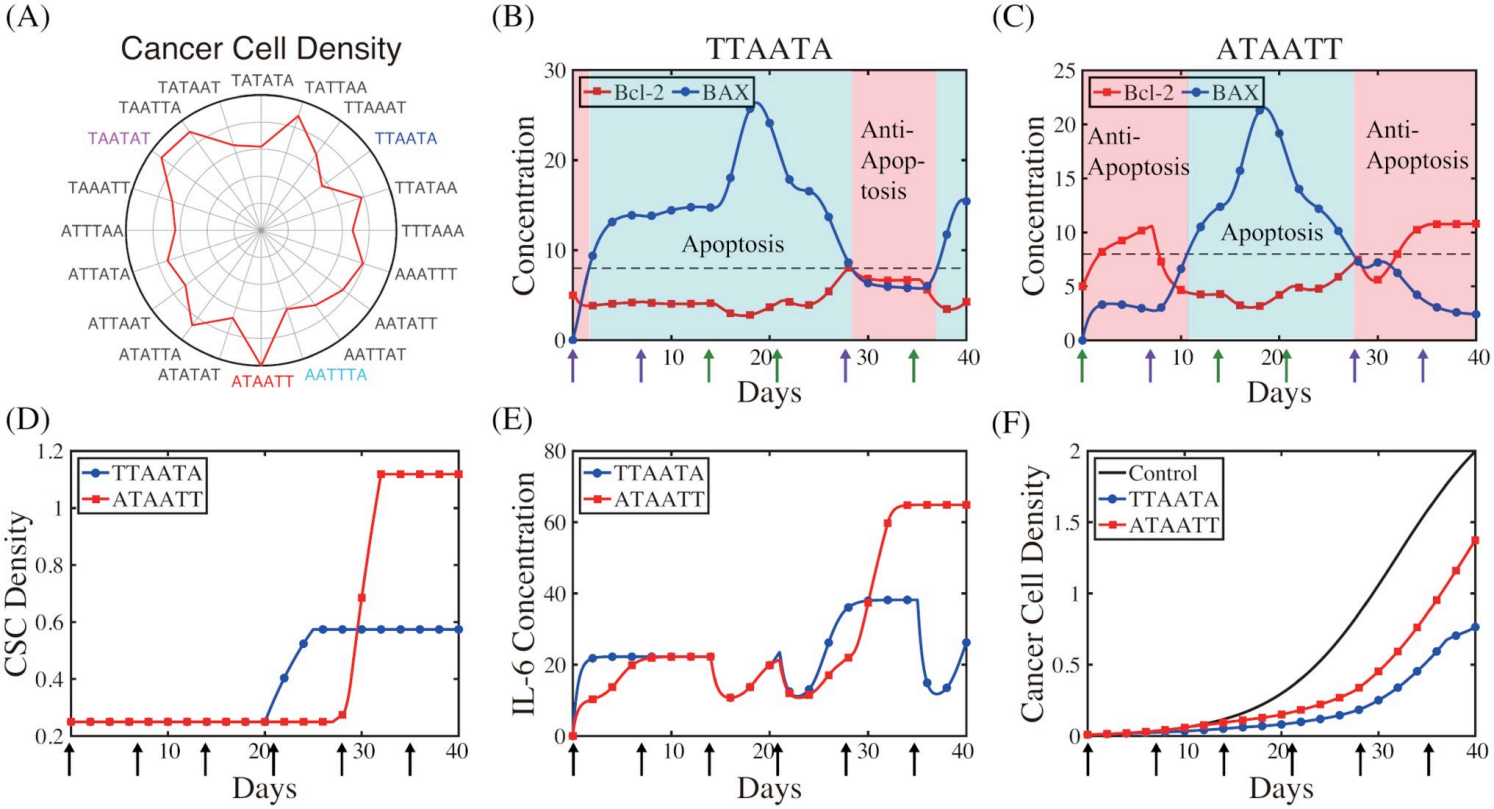
Optimized schedule: Maximizing anti-tumor efficacy. (A) Polar representation of the normalized cancer cell population at *t* = 40 *days* corresponding to the different alternating schemes of TZB and ATV. (B,C) Time courses of the Bcl-2 and BAX levels corresponding to the best (‘**TTAATA**’ in (B)) and worst (‘**ATAATT**’ in (C)) scheme. (D-F) Time courses of the CSC density (D), IL-6 concentration (E), and cancer cell density (F) corresponding to two schemes in (B,C). Parameters: *I*_*A*_ = 3.3, *I*_*T*_ = 0.075. Other parameters are given in Table 1.

**Fig 13:**
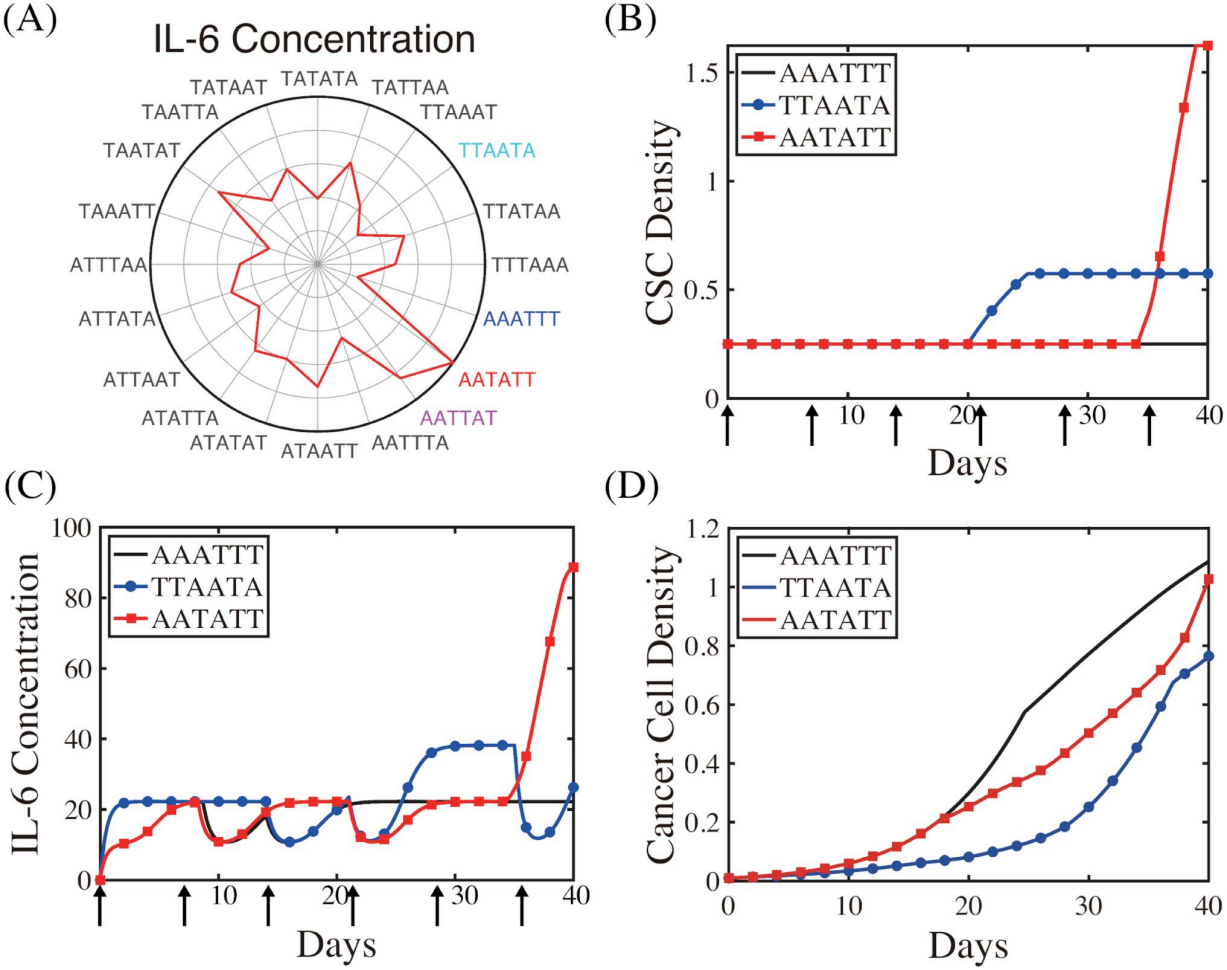
Optimized schedule: Preventing CRCC and minimizing anti-tumor efficacy. (A) Polar representation of the normalized IL-6 levels at *t* = 40 *days* corresponding to the different alternating schemes of TZB and ATV. (B-D) Time courses of the CSC density (B), IL-6 concentration (C), and cancer cell density (D) corresponding to the best (‘**AAATTT**’) and worst (‘**AATATT**’) scheme in preventing CRCC and the best (‘**TTAATA**’) scheme in minimizing both tumor volume and IL-6 level. Parameters: *I*_*A*_ = 3.3, *I*_*T*_ = 0.075. Other parameters are given in Table 1.

The injection scheme in the previous discussion shows various levels of anti-tumor efficacy and CRCC from multiple ATV infusion with fixed TZB supply. In this section, instead of periodic infusions of ATV and continuous administration of TZB, we assume three rounds of infusions for both anti-cancer agents and distribute them alternatively for the whole duration (40 *days*) of treatment at equally spaced (7 *days*) periods as in 17. We assume that ATV and TZB are injected over time intervals [*t*_*i*_, *t*_*i*_ + *h*_*A*_], i = 1,…,*N*_*A*_ and [*t*_*j*_, *t*_*j*_ + *h*_*T*_], i = 1,…,*N*_*T*_ with the time duration *h*_*T*_, respectively. Here, *N*_*A*_, *N*_*T*_ are the total number of ATV and TZB injections. For simulation, we introduce a new set of governing equations for temporal dynamics of ATV (*A*(*t*)) and TZB (*T*(*t*)) concentrations in addition to Eqs (7)–(14) as follows:

$$\begin{array}{ccc} & & \frac{dA}{dt}=\sum_{i=1}^{N_{A}}I_{A}J_{\left[t_{i},t_{i}+h_{A}\right]}-\mu_{A}A, \end{array}$$ (18)

$$\begin{array}{ccc} & & \frac{dT}{dt}=\sum_{j=1}^{N_{T}}I_{T}J_{\left[t_{j},t_{j}+h_{T}\right]}-\mu_{T}T, \end{array}$$ (19)

where *I*_*A*_, *I*_*T*_ are the ATV and TZB doses, respectively, and *μ*_*A*_, *μ*_*T*_ are the decay rates of ATV and TZB, respectively. Here, $J_{\left[t_{i},t_{i}+h_{A}\right]}$, $J_{\left[t_{j},t_{j}+h_{T}\right]}$ are indicator functions as before. In particular, we set *N*_*A*_ = *N*_*T*_ = 3, *I*_*A*_ = 3.3, *I*_*T*_ = 0.075 and each injection is administered at day 0, 7, 14, 21, 28 and 35. For example, consider an initial injection of ATV (‘**A**’), followed by three administrations of TZB (‘**TTT**’) and two infusions of ATV (‘**AA**’), then this scheme will be labelled as (‘**ATTTAA**’) (see Figs 12A and 13A). There are 20 different combinations of treatment assessed which scheme is the most effective and cost-efficient in anti-tumor efficacy and preventing CRCC.

The normalized cancer cell densities corresponding to these different schemes are visualized in Fig 12A where the centre in the circular representation indicates the 0 value and the far edge represents the 1 value. In addition, the length of the line connecting the centre and the final points represents the numerical values of the cancer cell population corresponding to each scheme. Thus, lower (or higher) cancer cell populations are represented by shorter (or longer) lines. Analysis suggests that combination ‘**ATAATT**’ is the least effective in cancer cell killing while the ‘**TTAATA**’ scheme is the most effective in eliminating cancer cells (Fig 12A and 12F). Wider ranges of apoptotic status and narrower strips of anti-apoptotic modes (Fig 12B) are observed in the best scheme (‘**TTAATA**’) in response to initial injection bundles of TZB at earlier time and regaining $T_{a}$-status from last ATV injection. On the contrary, in the worst scenario (‘**ATAATT**’), just one infusion of TZB as a second infusion at earlier time does not take full advantage of the strong anti-tumour activity from TZB, resulting in relatively slower entrance to the $T_{a}$-status (blue strip in Fig 12C) and intense injection of TZBs at later times only leads to emergence of $T_{t}$ due to onset of strong CSC responses (Fig 12D) at the late stage. This CSC reaction also stimulates IL-6 production (Fig 12E), increasing probability of CRCC in the worst scheme.

In Fig 13A we investigate the effect of combination therapy on onset of CRCC in response to various infusion schemes in Fig 12. Analysis suggests that the combination ‘**AATATT**’ is the least effective in lowering CSC activities (Fig 13B) and the IL-6 level (Fig 13C) while the ‘**AAATTT**’ scheme is the most effective in suppressing onset of CRCC. Overall, the scheme ‘**TTAATA**’ scheme provides the best strategies in maximizing the anti-tumor efficacy (Figs 12 and 13D) and minimizing the chances of CRCC (Fig 13C). Note that the IL-6 level immediately drops down in response to a ATV infusion but quickly recovers just before a succeeding ATV infusion (Fig 13C). In particular, it would be important to assign a ATV infusion at later times in order to effectively inhibit up-regulation of IL-6 levels (Fig 13C) and prevent onset of CRCC.

## Discussion

CRCC, cognitive impairments caused by chemical therapy, can affect up to 75% of cancer patients ((18–78)% of breast cancer patients [20, 68]) during treatment [69, 70] and became a new challenge as the number of long-term cancer survivors is rapidly increasing [25]. The difficulties in measuring CRCC contributes to the wide range of the percentage of cancer patients with cognitive impairments [71]. Cancer patients are exposed to CRCC after long term treatment of TZB, effective anti-cancer agent [72, 73]. *Apoptosis*, cellular death program, is an essential element of various processes, including proper development, homeostasis, and function of the immune system [74]. TZB induces cellular apoptosis of cancer cells by inhibiting Bcl-2 and regulating the downstream pathways [42, 75]. Unfortunately, long term administration of TZB can increase IL-6 levels through onset of CSCs, inducing a serious side effect, CRCC, and decrease anti-cancer efficacy by activating the NF-*κ*B signaling and downstream apoptotic pathways in cancer patients [35, 76].

The goal of this study is to identify fundamental mechanism of CRCC during TZB treatment and to develop key protocols of treatment combinations that induce both effective anti-tumour capabilities of anti-cancer agents and prevention of CRCC in cancer patients. In this work, we developed a mathematical model describing the dynamics of TZB and ATV in strategic treatment of tumour with CRCC. We investigated how TZB induces the apoptosis of cancer cells [75] by inhibiting Bcl-2 in the intracellular (NF-*κ*B, Bcl-2 and BAX) system (Figs 3 and 4) and how up-regulation of IL-6 from long term TZB treatment [22] can induce activation of NF-*κ*B which in turn induces up-regulation of Bcl-2 and down-regulation of BAX, switching the apoptotic mode to anti-apoptotic mode (Fig 4). ATV was shown to inhibit proliferation of breast cancer cells by inducing autophagy, another form of cell death program [77]. In particular, we showed that the combination therapy (TZB+ATV) can reduce IL-6 activities and changes the intracellular dynamics in the apoptosis pathway, turning it back to the apoptotic mode to the anti-apoptosis system (Figs 4 and 5). These results are in good agreement with experimental observation [10]. Owing to the complexity and vague mechanisms as well as a limitation of validated or approved tests due to the lack of sensitivity of assessment methods [65], the typical clinical approach is to refer cancer patients to psychiatrists who can order general coping strategies such as cognitive rehabilitation, mind-training exercises, changes to lifestyle, and cognitive-behavioral therapy [78–80] with supportive prescription of neuropsychiatric drugs [81]. Considering the complexity of applying new therapeutic compounds for the CNS [64] and convergent cellular mechanism of CRCC [66], our model suggests that prescription of ATV as a combination therapy can be an effective way of improving the cognitive level during TZB treatment (Fig 6). While the high dose of TZB is expected to gain best clinical outcomes, it causes unexpected side effects, CRCC, as well as lower anti-tumor efficacy, leading to the ‘V’-shape tumor size as a function of TZB doses (Fig 7D). The model suggests that while CRCC can be rescued by ATV combined with TZB (Fig 7E) in general, the best clinical outcome in both physical and mental health can be achieved by choosing an optimal, intermediate TZB dose in the combination therapy (Fig 7) with minimal costs (Figs 10, 12 and 13). An optimal control theory [82–85] can be applied to this complex system in brain in order to optimize the anti-tumor efficacy and minimize cognitive dysfunction with minimal costs.

In this work, we did not take into account other microenvironmental factors for CRCC in anti-cancer therapy such as signaling networks [86], neurogenesis and gliogenesis [66], biochemical interactions between cells such as microglia, neuron, and astrocytes [66], neuroimmune axes dynamics [52, 66] in the presence of the Blood-Brain Barriers [87]. A multi-scale mathematical model [5, 6, 48, 88–94] can be used to incorporate inter- and intra-cellular signaling at the microscale level and integrating biochemical and biomechanical mechanism of stromal cells and cancer cells at the cellular level. Further theoretical predictions and experimental validation of CRCC in cancer therapy need to be done as more experimental data are available. However, the mathematical model in this work may be a starting point of fundamental understanding of cognitive impairment in a destructive tumor microenvironment and further feedbacks from experimental investigation. We hope to address these detailed issues in near future.

## Supporting information

S1 Text Analysis of the intracellular model. (PDF)

S2 Text Parameter estimation and nondimensionalization. (PDF)

S3 Text Sensitivity analysis. (PDF)
