# Supplementary material for: Atorvastatin-mediated rescue of cancer-related cognitive changes in combined anticancer therapies
Source: PLoS Comput Biol. 2021 Oct 20;17(10):e1009457. doi: 10.1371/journal.pcbi.1009457 (PMC8559965; doi:10.1371/journal.pcbi.1009457)
Supplement: S1 Text — (PDF) [file pcbi.1009457.s001.pdf]

Supporting Information

Junho Lee, Jin Su Kim, Yangjin Kim

S1: Analysis of the intracellular model

1 Mathematical modeling of inhibition in the presence of autocatalytic activities

In this section, we describe the inhibition process of Bcl-2 and BAX levels in our modeling framework and take Bcl-2 as an example. The governing equation of the Bcl-2 concentration in the main text is given by

$$\frac{dB}{dt} = \lambda_B + \lambda_3 F + \frac{k_5 k_4^2}{k_4^2 + \delta F_1(T)} - \mu_B B, \tag{1}$$

where T is the TZB level and F is the NFκB concentration. In the absence of the 3rd term in Eq. (1), the Bcl-2 level always converges to the unique steady state $B^* = \frac{\lambda_B + \lambda_3 F}{\mu_B}$.

For the illustrative purpose, we take this equilibrium point as a baseline of the Bcl-2 level and focus on the effect of the inhibition process, 3rd term in Eq. (1), by considering the following simple equation

$$\frac{dB}{dt} = \frac{k_5 k_4^2}{k_4^2 + F(T)}. \tag{2}$$

Here, we set $F(T) = \delta F_1(T)$ and $F(T)$ is a time-dependent function, which describes the inhibition

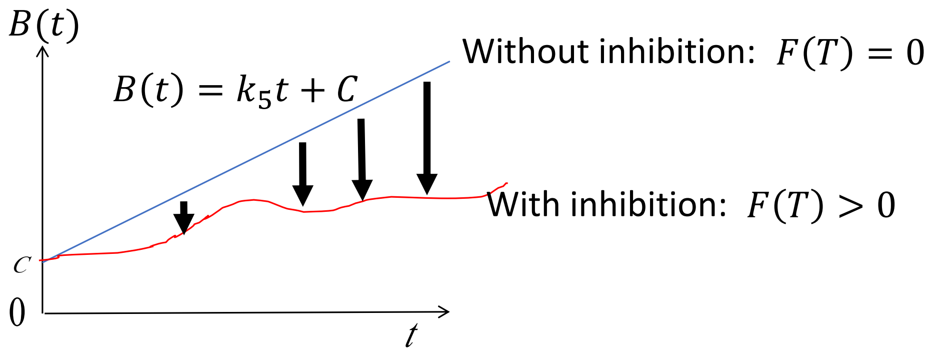

Figure S1. Effect of negative feedback loops on the Bcl-2 level in an ODE model in Eq. (2). Time courses of the Bcl-2 concentrations in the absence (blue) and presence (red) of the TZB-induced inhibition via the function $F(T)$.

of Bcl-2 level by the anti-cancer drugs, TZB (T). The general solution of Eq. (2) is expressed as $B(t) = k_5 k_4^2 \int_{t_0}^{t} \frac{1}{k_4^2 + F(T)} dt + C$ with an initial condition C. In the absence of inhibition ($F(T) = 0$), we get a simple, increasing function, $B(t) = k_5 t + C$. This implies the up-regulation of Bcl-2 concentration due to autocatalytic activities (increasing in time) in the absence of the negative feedback from other variables (blue curve in Fig. S1). On the other hand, the Bcl-2 level can be significantly down-regulated in the presence of the inhibition process (red curve in Fig. S1).

Fig. S2 shows time courses of the Bcl-2 concentration in the presence of various function forms $F(T)$. Here, we set $k_4 = k_5 = 1$ as in main text. When inhibition is strong ($F(T) = 99$ in Fig. S2A), the system bring the Bcl-2 level back to the baseline, leading to down-regulation. On the other hand, the Bcl-2 level can be significantly increased in response to a relatively weak negative feedback loop ($F(T) = e^{-t}$ in Fig. S2D). Strength and nonlinearity of the inhibition function $F(T)$ can induce strong ($F(T) = e^t$ in Fig. S2C) or intermediate level ($F(T) = t$ in Fig. S2B) of suppression.

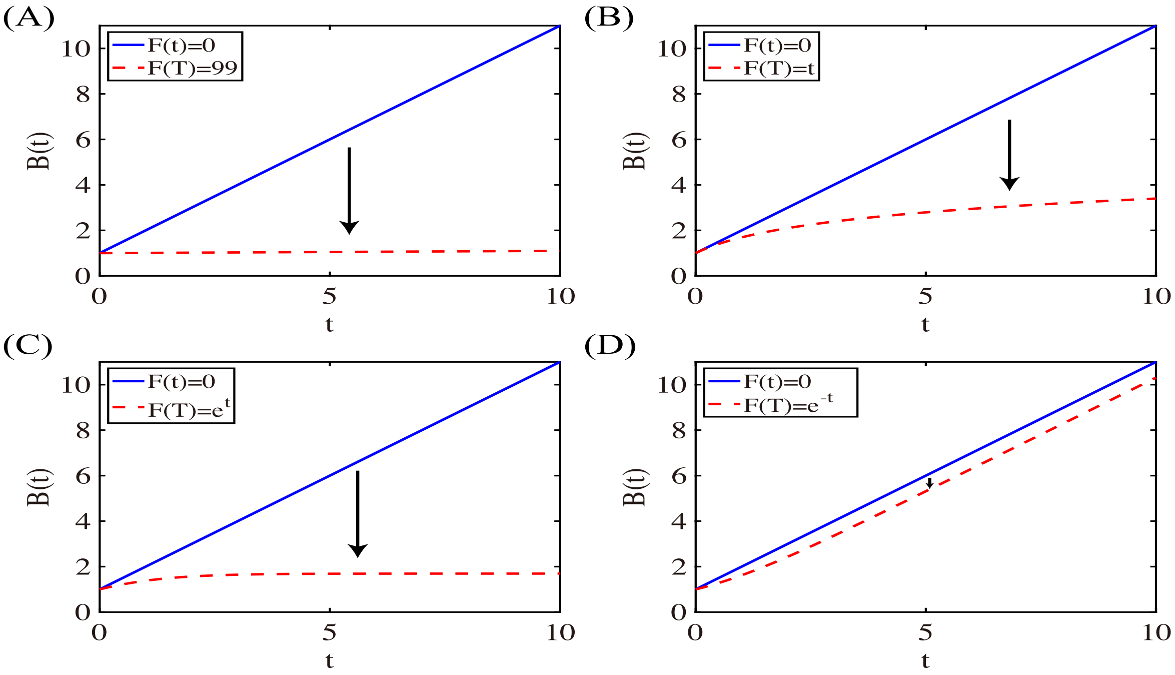

Figure S2. Changes in the Bcl-2 level in response to various inhibition functions in an ODE model in Eq. (2). Time courses of the Bcl-2 concentration in the presence (red dashed) and absence of inhibition (blue solid) for various functions $F(T)$: $F(T) = 99$ in(A); $F(T) = t$ in (B); $F(T) = e^t$ in (C); $F(T) = e^{-t}$ in (D). Here, $k_4 = k_5 = 1$.

2 Stability Analysis

The governing equations of the intracellular variables are

$$\frac{dF}{dt} = \lambda_F + \lambda_2 L - \mu_F F, \tag{3}$$

$$\frac{dB}{dt} = \lambda_B + \lambda_3 F + \frac{k_5 k_4^2}{k_4^2 + \delta T^2} - \mu_B B, \tag{4}$$

$$\frac{dX}{dt} = \lambda_X + \frac{k_7 k_6^2}{k_6^2 + \gamma B^2} - \mu_X X. \tag{5}$$

Steady states of the system (3)-(5) are given by

$$F^* = \frac{\lambda_F + \lambda_2 L}{\mu_F}, \tag{6}$$

$$B^* = \frac{1}{\mu_B}\left(\lambda_B + \lambda_3 F^* + \frac{k_5 k_4^2}{k_4^2 + \delta T^2}\right) = \frac{1}{\mu_B}\left(\lambda_B + \frac{\lambda_3(\lambda_F + \lambda_2 L)}{\mu_F} + \frac{k_5 k_4^2}{k_4^2 + \delta T^2}\right), \tag{7}$$

$$X^* = \frac{1}{\mu_X}\left(\lambda_X + \frac{k_7 k_6^2}{k_6^2 + \gamma {B^*}^2}\right) = \frac{1}{\mu_X}\left[\lambda_X + \frac{k_7 k_6^2}{k_6^2 + \frac{\gamma}{\mu_B}\left(\lambda_B + \frac{\lambda_3(\lambda_F+\lambda_2 L)}{\mu_F} + \frac{k_5 k_4^2}{k_4^2+\delta T^2}\right)^2}\right], \tag{8}$$

where $L = (\lambda_L + \frac{k_3 S}{k_1 + k_2 A})/\mu_L$.
The Jacobian matrix for at a steady state (F^*, B^*, X^*) is given by:

$$J = \begin{pmatrix} -\mu_F & 0 & 0 \\ \lambda_3 & -\mu_B & 0 \\ 0 & -2\gamma k_7 k_6^2 B/(k_6^2 + \gamma {B^*}^2)^2 & -\mu_X \end{pmatrix}.$$

Therefore, the lower triangle matrix J gives us three real negative eigenvalues ($-\mu_F < 0, \ -\mu_B < 0, \ -\mu_X < 0$), leading to *stable* equilibrium.
