# Supplementary material for: Atorvastatin-mediated rescue of cancer-related cognitive changes in combined anticancer therapies
Source: PLoS Comput Biol. 2021 Oct 20;17(10):e1009457. doi: 10.1371/journal.pcbi.1009457 (PMC8559965; doi:10.1371/journal.pcbi.1009457)
Supplement: S2 Text — (PDF) [file pcbi.1009457.s002.pdf]

Supporting Information

Junho Lee, Jin Su Kim, Yangjin Kim

S2: Parameter estimation and nondimensionalization

Nondimensionalization

The governing equations for main variables in the main text are as follows:

$$\frac{dF}{dt} = \lambda_F + \lambda_2 L - \mu_F F, \tag{1}$$

$$\frac{dB}{dt} = \lambda_B + \lambda_3 F + \frac{k_5 k_4^2}{k_4^2 + \delta T^2} - \mu_B B, \tag{2}$$

$$\frac{dX}{dt} = \lambda_X + \frac{k_7 k_6^2}{k_6^2 + \gamma B^2} - \mu_X X. \tag{3}$$

$$\frac{dC}{dt} = rC\left(1 - \frac{C}{K}\right) + \lambda_1 S - \mu_C \frac{\alpha}{B} C I_{apop}, \tag{4}$$

$$\frac{dS}{dt} = \beta T I_s, \tag{5}$$

$$\frac{dL}{dt} = \lambda_L + \frac{k_3 S}{k_1 + k_2 A} - \mu_L L. \tag{6}$$

$$\frac{dA}{dt} = \sum_{i=1}^{N_A} I_A J_{[t_i, t_i + h_A]} - \mu_A A, \tag{7}$$

$$\frac{dT}{dt} = \sum_{j=1}^{N_T} I_T J_{[t_j, t_j + h_T]} - \mu_T T, \tag{8}$$

We non-dimensionalize the variables and the parameters in Eqs (1)-(8) as follows

$$\begin{aligned}
&\bar{t} = \frac{t}{t^*},\ \bar{F} = \frac{F}{F^*},\ \bar{B} = \frac{B}{B^*},\ \bar{X} = \frac{X}{X^*},\ \bar{C} = \frac{C}{C^*},\ \bar{S} = \frac{S}{S^*},\ \bar{L} = \frac{L}{L^*},\ \bar{T} = \frac{T}{T^*},\ \bar{A} = \frac{A}{A^*},\\
&\bar{\lambda}_F = \frac{t^*\lambda_F}{F^*},\ \bar{\lambda}_2 = \frac{t^* L^* \lambda_2}{F^*},\ \bar{\mu}_F = t^*\mu_F,\ \bar{\lambda}_B = \frac{t^*\lambda_B}{B^*},\ \bar{\lambda}_3 = \frac{t^* F^* \lambda_3}{B^*},\ \bar{k}_4 = k_4,\\
&\bar{k}_5 = \frac{t^* k_5}{B^*},\ \bar{\delta} = {T^*}^2\delta,\ \bar{\mu}_B = t^*\mu_B,\ \bar{\lambda}_X = \frac{t^*\lambda_X}{X^*},\ \bar{k}_6 = k_6,\ \bar{k}_7 = \frac{t^* k_7}{X^*},\ \bar{\gamma} = {B^*}^2\gamma,\\
&\bar{\mu}_X = t^*\mu_X,\ \bar{r} = t^* r,\ \bar{K} = \frac{K}{C^*},\ \bar{\lambda}_1 = \frac{t^* S^* \lambda_1}{C^*},\ \bar{\mu}_C = t^*\mu_C,\ \bar{\alpha} = \frac{\alpha}{B^*},\ \bar{\beta} = \frac{t^* T^* \beta}{S^*},\\
&\bar{\lambda}_L = \frac{t^*\lambda_L}{L^*},\ \bar{k}_1 = k_1,\ \bar{k}_2 = k_2 A^*, \bar{k}_3 = \frac{t^* S^* k_3}{L^*},\ \bar{\mu}_L = t^*\mu_L,\ \bar{I}_A = \frac{t^* I_A}{A^*},\ \bar{\mu}_A = t^*\mu_A,\\
&\bar{I}_T = \frac{t^* I_T}{T^*},\ \bar{\mu}_T = t^*\mu_T,
\end{aligned} \tag{9}$$

where the characteristic time t^* and reference values $F^*, B^*, X^*, C^*, S^*, L^*, T^*, A^*$ are given in Table S1.

Then, the governing equations in a dimensionless form are

$$\frac{d\overline{F}}{d\overline{t}} = \overline{\lambda}_F + \overline{\lambda}_2\overline{L} - \overline{\mu}_F\overline{F}, \tag{10}$$

$$\frac{d\overline{B}}{d\overline{t}} = \overline{\lambda}_B + \overline{\lambda}_3\overline{F} + \frac{\overline{k}_5\overline{k}_4^2}{\overline{k}_4^2 + \overline{\delta T}^2} - \overline{\mu}_B\overline{B}, \tag{11}$$

$$\frac{d\overline{X}}{d\overline{t}} = \overline{\lambda}_X + \frac{\overline{k}_7\overline{k}_6^2}{\overline{k}_6^2 + \overline{\gamma B}^2} - \overline{\mu}_X\overline{X}. \tag{12}$$

$$\frac{d\overline{C}}{d\overline{t}} = \overline{r}\overline{C}\left(1 - \frac{\overline{C}}{\overline{K}}\right) + \overline{\lambda}_1\overline{S} - \overline{\mu}_C\frac{\overline{\alpha}}{\overline{B}}\overline{C}I_{apop}, \tag{13}$$

$$\frac{d\overline{S}}{d\overline{t}} = \overline{\beta T}I_s, \tag{14}$$

$$\frac{d\overline{L}}{d\overline{t}} = \overline{\lambda}_L + \frac{\overline{k}_3\overline{S}}{\overline{k}_1 + \overline{k}_2\overline{A}} - \overline{\mu}_L\overline{L}, \tag{15}$$

$$\frac{d\overline{A}}{d\overline{t}} = \sum_{i=1}^{N_A}\overline{I}_A J_{[t_i,t_i+h_A]} - \overline{\mu}_A\overline{A}, \tag{16}$$

$$\frac{d\overline{T}}{d\overline{t}} = \sum_{j=1}^{N_T}\overline{I}_T J_{[t_j,t_j+h_T]} - \overline{\mu}_T\overline{T}. \tag{17}$$

Table S1. Reference variables used in the model.

	Description	Dimensional Value	Refs.
t^*	Time	$1\ h$	
C^*	Cancer density	10^6 cells/mm^3	[1–4]
S^*	Cancer stem cell density	10^3 cells/mm^3	[5]
L^*	IL-6 concentration	16.4 pg/mL	[6]
F^*	NF-κB concentration	0.5 μM	[1, 7–10]
B^*	Bcl-2 concentration	0.5 μM	[11]
X^*	BAX concentration	0.1 μM	[1, 12]
T^*	Trastuzumab concentration	5 $\mu g/mL$	[13]
A^*	Atorbastatin concentration	20 μM	[13]

Parameter Estimation

Dimensional values of the various parameters of the mathematical model system (1)-(6) are provided in Table 1 in the main text.
The decay rate (μ) of a molecule can be calculated by its half life ($T_{1/2}$): $\mu = \frac{ln(2)}{T_{1/2}}$.

μ_F, μ_B, μ_X (decay rates of NF-κB, Bcl-2 and BAX): Half-life of suppressor of NF-κB, IκB, is reported to be in the range of (10-40) *mins* [14–16]. By taking 40 *mins* of half-life of IκB and 2.2 *hours* of half-life for the NFκB [1], we get the decay rate of IκB, $\mu_s = ln(2)/(40\ min) = 1.0\ h^{-1}$, and decay rate of NF$\kappa$B,

$\mu_F = 3 \times 10^{-1}\ h^{-1}$. Half-life of Bax is reported to be in the range of (6-32) *hours* [17], leading to the possible decay rates of $\frac{ln(2)}{(32\ h)} - \frac{ln(2)}{(6\ h)} = (0.0217 - 0.1155)\ h^{-1}$. We take $\mu_X = 2 \times 10^{-2}\ h^{-1}$. Half-life of Bcl-2 was reported to be 20 *hours* [18], leading to $\mu_B = \frac{ln(2)}{20h} = 3.47 \times 10^{-2}\ h^{-1}$.

μ_L (decay rate of IL-6): The half life of IL-6 was reported to be in the range of 2-15.5 hours [19, 20]. By taking 8.5 hours, we get the decay rate $\mu_L = ln(2)/(8.5\ h) = 0.0815\ h^{-1}$.

μ_A (decay rate of Atorvastatin): The half life of ATV in human was reported to be 14 h [21], leading to the decay rate $\mu_A = ln(2)/(14\ h) = 0.0495\ h^{-1}$.

μ_T (decay rate of TZB): The half life of TZB was reported to be 5.8 *day* [22], leading to the decay rate $\mu_T = ln(2)/(139\ h) = 5.0 \times 10^{-3}\ h^{-1}$.

References

1. Kim Y, Lee J, Lee D, Othmer HG. Synergistic Effects of Bortezomib-OV Therapy and Anti-Invasive Strategies in Glioblastoma: A Mathematical Model. Cancers. 2019;11(2):E215.

2. Kim Y, Yoo JY, Lee TJ, Liu J, Yu J, Caligiuri MA, et al. Complex role of NK cells in regulation of oncolytic virus-bortezomib therapy. Proc Natl Acad Sci U S A. 2018;115(19):4927–32.

3. Friedman A, Tian JP, Fulci G, Chiocca EA, Wang J. Glioma virotherapy: effects of innate immune suppression and increased viral replication capacity. Cancer Res. 2006;66(4):2314–9.

4. Othmer HG, Xie M. Implicit and Explicit Methods for Excitable Systems. Technical Report. 2006;.

5. Reya T, Morrison SJ, Clarke MF, Weissman IL. Stem cells, cancer, and cancer stem cells. Nature. 2001;414(6859):105–11.

6. Khan A, Ali Z. Normal Ranges for Acute Phase Reactants (Interleukin-6, Tumour Necrosis Factor-alpha and C-reactive Protein) in Umbilical Cord Blood of Healthy Term Neonates at the Mount Hope Women's Hospital, Trinidad. West Indian Med J. 2014;63(5):465–9.

7. Lipniacki T, Paszek P, Brasier AR, Luxon B, Kimmel M. Mathematical model of NF-kappaB regulatory module. J Theor Biol. 2004;228(2):195–215.

8. Lee EG, Boone DL, Chai S, Libby SL, Chien M, Lodolce JP, et al. Failure to regulate TNF-induced NF-kappaB and cell death responses in A20-deficient mice. Science. 2000;289(5488):2350–4.

9. Mothes J, Busse D, Kofahl B, Wolf J. Sources of dynamic variability in NF-kB signal transduction: A mechanistic model. Bioessays. 2015;37(4):452–62.

10. Xue X, Xia W, Wenzhong H. A modeled dynamic regulatory network of NF-κB and IL-6 mediated by miRNA. Biosystems. 2013;114(3):214–8.

11. Placzek WJ, Wei J, Kitada S, Zhai D, Reed JC, Pellecchia M. A survey of the anti-apoptotic Bcl-2 subfamily expression in cancer types provides a platform to predict the efficacy of Bcl-2 antagonists in cancer therapy. Cell Death Dis. 2010;1:e40.

12. Kirkland RA, Saavedra GM, Cummings BS, Franklin JL. Bax regulates production of superoxide in both apoptotic and nonapoptotic neurons: role of caspases. J Neurosci. 2010;30(48):16114–27.

13. Lee S, Lee HJ, Kang H, Kim EH, Lim YC, Park H, et al. Trastuzumab Induced Chemobrain, Atorvastatin Rescued Chemobrain with Enhanced Anticancer Effect and without Hair Loss-Side Effect. J Clin Med. 2019;8(2):E234.

14. Bergqvist S, Ghosh G, Komives EA. The IkBa/NF-kB complex has two hot spots, one at either end of the interface. Protein Science. 2008;17:2051–2058.

15. Mathes E, O?Dea EL, Hoffmann A, Ghosh G. NF-kB dictates the degradation pathway of IkBa. The EMBO Journal. 2008;27:1357–1367.

16. Krappmann D, Scheidereit C. Regulation of NF-kappa B activity by I kappa B alpha and I kappa B beta stability. Immunobiology. 1997;198(1-3):3–13.

17. Xin M, Deng X. Nicotine Inactivation of the Proapoptotic Function of Bax through Phosphorylation. THE JOURNAL OF BIOLOGICAL CHEMISTRY. 2005;280(11):10781–10789.

18. Rooswinkel RW, van de Kooij B, de Vries E, Paauwe M, Braster R, Verheij M, et al. Antiapoptotic potency of Bcl-2 proteins primarily relies on their stability, not binding selectivity. Blood. 2014;123(18):2806–15.

19. Waage A, Brandtzaeg P, Halstensen A, Kierulf P, Espevik T. The complex pattern of cytokines in serum from patients with meningococcal septic shock. Association between interleukin 6, interleukin 1, and fatal outcome. J Exp Med. 1989;169(1):333–8.

20. Kuribayashi T. Elimination half-lives of interleukin-6 and cytokine-induced neutrophil chemoattractant-1 synthesized in response to inflammatory stimulation in rats. Lab Anim Res. 2018;32(2):80–3.

21. McIver LA, Siddique MS. Atorvastatin. StatePearls Publishing; 2019.

22. Goldenberg MM. Trastuzumab, a recombinant DNA-derived humanized monoclonal antibody, a novel agent for the treatment of metastatic breast cancer. Clin Ther. 1999;21(2):309–18.
