# Supplementary material for: Atorvastatin-mediated rescue of cancer-related cognitive changes in combined anticancer therapies
Source: PLoS Comput Biol. 2021 Oct 20;17(10):e1009457. doi: 10.1371/journal.pcbi.1009457 (PMC8559965; doi:10.1371/journal.pcbi.1009457)
Supplement: S3 Text — (PDF) [file pcbi.1009457.s003.pdf]

Supporting Information

Junho Lee, Jin Su Kim, Yangjin Kim

S3: Sensitivity Analysis

Par / PRCC	λ_2	δ	γ	r	λ_1	μ_C	k_2	k_3
$C(24)$	0.0456**	-0.0468**	0.1852**	0.8871**	0.7668**	-0.1644**	-0.0267**	0.0231*
$L(24)$	-0.0044	0.0101	-0.0274**	-0.0067	0.0052	0.0111	-0.9694**	0.9818**
$F(24)$	0.9723**	0.0046	-0.0033	-0.0065	0.0013	0.0038	-0.7889**	0.8616**
$B(24)$	0.8768**	-0.7623**	-0.0026	-0.0044	-0.0008	0.0041	-0.5086**	0.6177**
$X(24)$	-0.7932**	0.7220**	-0.9864**	-0.0023	0.0021	0.0149	0.4545**	-0.5607**
$C(480)$	0.4635**	-0.2981**	0.2184**	0.8827**	0.4405**	-0.3797**	-0.1443**	0.1989**
$L(480)$	-0.0043	0.0101	-0.0274**	-0.0067	0.0052	0.0111	-0.9694**	0.9818**
$F(480)$	0.9723**	0.0046	-0.0033	-0.0065	0.0013	0.0039	-0.7889**	0.8616**
$B(480)$	0.9169**	-0.6731**	-0.0027	-0.0052	-0.0016	0.0036	-0.5782**	0.6862**
$X(480)$	-0.8972**	0.6281**	-0.8544**	0.0050	0.0044	0.0107	0.5532**	-0.6638**
$C(960)$	0.5218**	-0.3183**	0.2654**	0.8408**	0.3786**	-0.3449**	-0.1507**	0.2269**
$L(960)$	-0.0043	0.0101	-0.0274**	-0.0067	0.0052	0.0111	-0.9694**	0.9818**
$F(960)$	0.9723**	0.0046	-0.0033	-0.0065	0.0013	0.0038	-0.7889**	0.8616**
$B(960)$	0.9169**	-0.6731**	-0.0027	-0.0052	-0.0016	0.0036	-0.5782**	0.6862**
$X(960)$	-0.8971**	0.6281**	-0.8544**	0.0050	0.0044	0.0107	0.5532**	-0.6638**
Min	0.003	1	0.2	7×10^{-4}	5×10^{-6}	3×10^{-4}	0.1	0.4
Base	0.03	72	2	7×10^{-3}	5×10^{-5}	3×10^{-3}	1	4
Max	0.3	300	20	7×10^{-2}	5×10^{-4}	3×10^{-2}	10	40

Table S1. Sensitivity analysis for the local ODE system at time t=24, 480, 960. Parameters used in sensitivity analysis and PRCC values of populations of cancer cells (C) and concentration of IL-6, NF-κB, Bcl-2 and BAX (L, F, B, X) at various time points ($t = 24, 480, 960$) are shown for 8 perturbed parameters $\lambda_2, \delta, \gamma, r, \lambda_1, \mu_C, k_2, k_3$. A range (minimum/maximum) of these 8 perturbed (non-dimensional) parameters are given in the lower section. Sample size=10000. *Significant (p-value < 0.05). **Significant (p-value < 0.01).
